# Learning endometriosis phenotypes from patient-generated data

**DOI:** 10.1038/s41746-020-0292-9

**Published:** 2020-06-24

**Authors:** Iñigo Urteaga, Mollie McKillop, Noémie Elhadad

**Affiliations:** 1 Department of Applied Physics and Applied Mathematics, Columbia University, New York, NY 10027 USA; 2 Data Science Institute, Columbia University, New York, NY 10027 USA; 3 Department of Biomedical Informatics, Columbia University, New York, NY 10032 USA

**Keywords:** Experimental models of disease, Chronic pain, Computational science, Reproductive signs and symptoms, Statistics

## Abstract

Endometriosis is a systemic and chronic condition in women of childbearing age, yet a highly enigmatic disease with unresolved questions: there are no known biomarkers, nor established clinical stages. We here investigate the use of patient-generated health data and data-driven phenotyping to characterize endometriosis patient subtypes, based on their reported signs and symptoms. We aim at unsupervised learning of endometriosis phenotypes using self-tracking data from personal smartphones. We leverage data from an observational research study of over 4000 women with endometriosis that track their condition over more than 2 years. We extend a classical mixed-membership model to accommodate the idiosyncrasies of the data at hand, i.e., the multimodality and uncertainty of the self-tracked variables. The proposed method, by jointly modeling a wide range of observations (i.e., participant symptoms, quality of life, treatments), identifies clinically relevant endometriosis subtypes. Experiments show that our method is robust to different hyperparameter choices and the biases of self-tracking data (e.g., the wide variations in tracking frequency among participants). With this work, we show the promise of unsupervised learning of endometriosis subtypes from self-tracked data, as learned phenotypes align well with what is already known about the disease, but also suggest new clinically actionable findings. More generally, we argue that a continued research effort on unsupervised phenotyping methods with patient-generated health data via new mobile and digital technologies will have significant impact on the study of enigmatic diseases in particular, and health in general.

## Introduction

Endometriosis is a chronic and systemic disease in women of reproductive age with no known cure^1–3^. Although complex multi-factorial causes (i.e., biological and environmental factors) are likely to be of relevance, the etiology of the disease is still unknown. Disease pathology is traditionally described by tissue similar to the endometrium—the lining of the uterus—growing outside the uterine cavity, which may form lesions in pelvic, gastrointestinal, and other areas. The disease is currently diagnosed by direct visualization of such lesions through laparoscopic surgery.

Endometriosis is prevalent in women, with estimates of affecting 10% of those in reproductive age, and has high morbidity and impact on quality of life^4,5^. Nevertheless, it is a highly enigmatic condition, with heterogeneous symptoms documented by patients: stereotypical evidence like pain and infertility are known, but a wide range of other symptoms with systemic effects are reported as well^6^. However, these variety of symptoms have not been well characterized yet for all endometriosis patients, with unclear associations between some symptoms and the disease: it is still uncertain why some treatments are effective for some patients, and not for others. Besides, there are no known biomarkers of the disease for non-invasive diagnosis or for monitoring its progression, and it currently takes an average of 8 years for patients to receive a diagnosis. Although several stages of the disease have been proposed, they do not explain the diversity of symptoms experienced by patients, they do not correlate with their severity^7^, nor have unequivocal connection with disease progression^8^.

Due to its poor clinical characterization, identifying signatures across individuals that correspond to phenotypes of endometriosis would allow for better treatment, as well as to generate new hypotheses about potential causes and means of diagnosis^9^. An accurate characterization of endometriosis through disease subtypes is critical for earlier diagnosis, as well as for targeted treatment and management strategies of the disease. Traditional clinical phenotyping approaches based on available electronic health record data are limited, mostly due to the lack of sufficient evidence of the symptomatic manifestations of the disease. Furthermore, there is no existing grouping for characterization of the disease in the context of non-clinical, but easily observable, variables: e.g., signs and symptoms, such as pelvic pain, mood variations, or period characteristics, experienced by patients.

Recently, wearable sensors^10,11^ and smartphones^12,13^ have been proposed as a powerful way to connect medical researchers to patients, and vice versa. With these mobile technologies, patients can provide longitudinal, real-world evidence of their experience of a particular disease. Recent software platforms like ResearchKit^14^ and ResearchStack^15^ facilitate the use of mobile technology to recruit and consent patients into studies^16^. The first wave of app-based studies have shown that patients can provide valuable information, with the appropriate recruitment and retention strategies^17^, to advance our understanding of disorders over time, generating new insights about diseases^18,19^ and overall health^20,21^.

This work contributes to the emerging area of research on digital phenotyping from patient-generated health data, specifically from data collected through smartphone applications^13,22^. Digital phenotyping aims at the automatic characterization of a patient’s phenotype using electronic data. In conjunction with the advance of data science and machine learning techniques, along with the pervasive use of smartphones, other personal digital devices and wearables, it holds considerable potential for analyzing patient-generated data^20,23^ for medical research purposes^12,13,16,24–27^.

In this work, we explore the use of unsupervised data-driven methods to identify subtypes of endometriosis, where patients are grouped together based on their signs and symptoms, quality of life, and treatments. We use self-tracking data obtained through an smartphone app specifically designed to characterize endometriosis at scale. We extend a mixed-membership model—which partitions collections of data into mixtures of a shared set of latent groups—to accommodate the idiosyncrasies of the data at hand: i.e., the multimodality and uncertainty of the tracked variables. We probabilistically model a wide range of observations (i.e., participant symptoms, quality of life, treatments) to obtain interpretable descriptions of endometriosis phenotypes.

We validate our approach both intrinsically and extrinsically via (1) the evaluation of its ability to model unseen data, (2) the interpretability of the identified subtypes by endometriosis experts, (3) the matching of unsupervised phenotype assignments against clinical experts grouping, and (4) the association between subtypes and responses to clinically validated standard surveys for endometriosis.

Our experiments show that (i) our approach identifies phenotypes that are robust to biases of self-tracked data (e.g., wide variations in tracking frequency amongst participants), as well as to hyperparameter choices for the model; and (ii) jointly modeling a wide range of observations self-tracked by participants (symptoms, quality of life, treatments) yields clinically meaningful disease subtypes, both validating what is already known about endometriosis and suggesting new hypothesis about the condition as well. Overall, we show the promise of unsupervised learning of endometriosis phenotypes from self-tracked participant data collected via digital mobile platforms.

## Results

### Patient-generated data

We collected two types of patient-generated data for this study. Once participants consented, they were asked to self-track their symptoms in the Phendo research app, as well as to fill out an electronic version of the WERF survey, a validated clinical survey by and for the endometriosis research community. The unsupervised phenotype learning task relied only on the self-tracking data from Phendo, while the WERF survey data was used to assess the quality of the learned phenotypes.

### Patient-generated data—Phendo self-tracking data

**Fig. 1:**
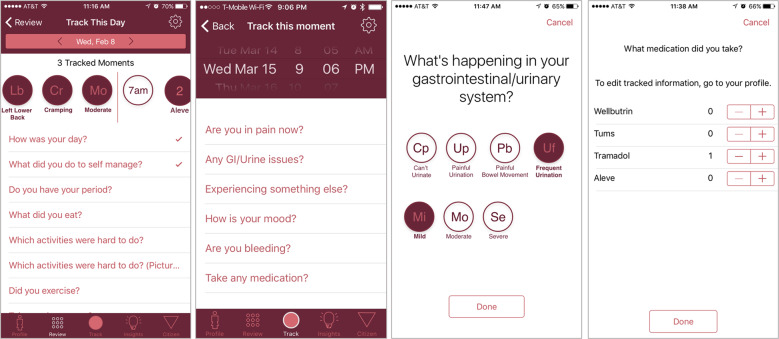
Example screenshots of Phendo, the endometriosis research app. Participants can answer multiple questions (e.g., related to gastrointestinal and genitourinary issues above) by selecting from a set of answers (e.g., “*painful urination*” or “*frequent urination*”).

Phendo is a Columbia University IRB-approved smartphone app for women to self-track endometriosis (Fig. 1), available for both iOS^28^ and Android^29^ based phones. The app was specifically designed to capture the patient experience of the disease, as well as to engage participants in self-tracking the condition over time^30,31^. App users were recruited through patient advocacy groups, and active recruitment efforts were sustained throughout the study period, leveraging a wide range of strategies including social media (Twitter, Facebook, Instagram, and Medium), emails, radio, news articles, celebrity endorsement through social media posts, blog posts, and scientific articles.

Once enrolled in the Phendo study, users can self-track a variety of variables of their interest at the frequency with which they experience them. Some—pain for example—moment-by-moment (i.e., when and as many times as participants experience it), while others—like “*How was your day?*”—are tracked daily. The app is purposely designed with these flexible options to collect data as close in time as to when the relevant events occur.

The moment-level tracking comprises reports about pain across specific body locations and severity levels, gastrointestinal and genitourinary issues relevant for endometriosis—with their associated severity levels—other signs and symptoms commonly reported by participants (e.g., “*blurry vision*”, “*hot flashes*”, “*fatigue*”) and their severity, participants’ bleeding patterns, and customized medication and hormonal intake reports. Users can track a functional assessment of their day (from “*Great*” to “*Unbearable*”), which daily living activities were hard for them to do, menstruation patterns, sexual activity and potential dyspareunia, as well as other personalized answers for hormonal treatments, diet and exercise items they want to keep track of.

We selected a cohort of Phendo participants who had self-reported diagnosis of endometriosis, and had at least one self-tracked entry in one of the available questions between December 2016 (launch of the app) and end of December 2018, resulting in 4368 participants—mostly white and non-hispanic, with a mean age of 29 (see Table 1 for the cohort characteristics).

**Table 1:** Phendo cohort (*N* = 4368) demographics.

Phendo cohort demographics	
Demographic variable	N (%) or Mean (s.d.)
Age	
Mean (s.d.)	30.29 (7.0)
Gender	*N* = 4351
Male	3 (0.1%)
Other	40 (0.9%)
Female	4308 (99.0%)
BMI	*N* = 4195
Underweight	201 (4.8%)
Obese	1979 (47.2%)
Normal	2015 (48.0%)
Race/ethnicity	*N* = 4350
Native american	29 (0.7%)
Black, non-hispanic	101 (2.3%)
Asian	111 (2.6%)
Hispanic	215 (4.9%)
Other	290 (6.7%)
White, non-hispanic	3604 (82.9%)
Education	*N* = 4348
High-school or under	639 (14.7%)
Some college	1320 (30.4%)
More than college	2389 (55.9%)
Living environment	*N* = 4344
Rural	718 (16.5%)
Urban	1755 (40.4%)
Suburban	1871 (43.1%)

In this study, we focused on the following subset of questions related to: (1) pain location with 39 potential answers, (2) pain description with 15 potential answers, (3) pain severity with 3 potential answers, (4) gastrointestinal and genitourinary (GI/GU) symptoms with 14 potential answers, (5) their severity with 3 potential answers, (6) other symptoms with 21 potential answers, (7) their severity with 3 potential answers, (8) period flow with 3 potential answers, (9) bleeding patterns with 3 potential answers, (10) sexual activity with 6 potential answers, (11) difficult daily living activities with 23 potential answers, (12) medications including hormonal treatments with 64 potential answers, and (13) quality of life with 5 potential answers. The details for the potential answers per-question are provided in the Supplementary Results.

Since the Phendo data (with 776,855 observations in total for the cohort) are self-tracked at the participants’ discretion, they are heterogeneous both in their frequency and their amounts collected per participant. The aggregated statistics over all the observations per tracked variable are described in Table 2.

**Table 2:** Summary statistics per-tracked question.

Summary statistics per-tracked question		
Question	Number of observations (mean/max)	Number of tracked days (mean/max)
Where is the pain?	31/2382	6/245
Describe the pain.	29/1745	6/245
How severe is the pain?	10/803	6/245
What are you experiencing?	9/907	4/188
How severe is the symptom?	5/552	4/188
Describe your period flow.	3/243	3/243
What kind of bleeding.	2/173	2/77
Describe GI/GU system.	7/342	4/205
How severe is it?	6/321	4/205
Describe sex.	1/203	1/195
Activities difficult to perform.	42/2148	7/404
How was your day?	13/710	13/710
Medications/hormones taken.	15/1623	9/368
Total	177/8253	76/2523

The lowest number of responses for the sex-related question occurs because it is not a default question in the Phendo app. Users must go to the app settings and specifically add this question to their daily tracked variables. The app is designed this way because individuals 13 and older are eligible to participate.

### Patient-generated data—WERF survey data

The WERF EPHect survey is a standardized questionnaire designed by the endometriosis research community^32^, and it represents the gold-standard for clinical characterization of endometriosis. The survey was optional for our study participants, and it was provided as part of the profile tab in the Phendo app. We selected a subset of questions related to menstrual and endometriosis history, family history of endometriosis, family history of chronic pelvic pain, and surgical history (Table 3), as well as diagnosed comorbidities, general health and activities of daily living (Table 4) for our analysis. Of the 4368 participants who contributed self-tracking data, 533 participants completed the WERF survey.

**Table 3:** WERF survey statistics for participants’ medical history (*N* = 533).

WERF survey statistics for participants’ medical history	
WERF Survey question	*N* (%)
Menstrual and endometriosis history	
Age at menarchy: Mean (s.d.)	12.13 (1.6)
Were your periods in the last 3 months hormone-induced?	165 (31.0%)
Were your periods in the the last 3 months regular?	239 (44.8%)
How many days of bleeding did you usually have for each period in the last 3 months (Not counting discharge/spotting for which you needed a panty liner only)? Mean (s.d.)	6.24 (3.6)
At what age did you start having pain with your period? Mean (s.d.)	14.63 (4.7)
How old were you when you first had symptoms? Mean (s.d.)	17.79 (6.9)
How many doctors did you see before receiving a diagnosis of endometriosis? Mean (s.d.)	5.03 (4.6)
How many surgical procedures have you had for endometriosis or pelvic pain? Mean (s.d.)	1.70 (1.6)
Have you ever had surgery to look for endometriosis and none was found?	45 (8.6%)
Family history of endometriosis	202 (37.9%)
Mother	92 (17.3%)
Daughter	1 (0.2%)
Sister	32 (6.0%)
Maternal Grandmother, aunt, and/or cousin	86 (16.1%)
Paternal Grandmother, aunt, and/or cousin	63 (11.8%)
Family history of chronic pelvic pain	240 (45.0%)
Mother	136 (25.5%)
Daughter	4 (0.8%)
Sister	61 (11.4%)
Maternal Grandmother, aunt, and/or cousin	107 (20.1%)
Paternal Grandmother, aunt, and/or cousin	73 (13.7%)
Surgical history	
Appendectomy	89 (16.7%)
Hysterectomy	22 (4.1%)
Oophorectomy	22 (4.1%)
Cervical surgery (LEEP or conization)	21 (3.9%)
Hysteroscopy	71 (13.3%)
Gallbladder surgery	35 (6.6%)
Hernia operation	21 (3.9%)
Sigmoidoscopy/colonoscopy	118 (22.1%)
Laparoscopy count	1.41 (1.3)
Some abdominal surgery	417 (78.2%)

Note that not all Phendo participants completed the WERF survey.

**Table 4:** WERF survey statistics for participants’ comorbidities (*N* = 533).

WERF survey statistics for participants’ comorbidities	
Diagnosed comorbidities	N (%)
Irritable-bowel syndrome	136 (25.5%)
Hashimoto’s disease	16 (3.0%)
Graves’ disease	3 (0.6%)
Glandular Fever	38 (7.1%)
Fibromyalgia	27 (5.1%)
Anxiety disorder requiring medication or therapy	261 (49.0%)
Asthma	151 (28.3%)
Cardiovascular disease	14 (2.6%)
Some cancer	14 (2.6%)
Crohn’s disease	4 (0.8%)
Chronic fatigue syndrome	31 (5.8%)
Deaf/difficulty hearing	6 (1.1%)
Depression/mood disorder requiring medication or therapy	274 (51.4%)
Diabetes	5 (0.9%)
Uterine fibroids	70 (13.1%)
High blood pressure	33 (6.2%)
Migraine	203 (38.1%)
Mitral valve prolapse	7 (1.3%)
Multiple sclerosis	1 (0.2%)
Painful bladder/interstitial cystitis (NOT bacterial bladder infection)	36 (6.8%)
Pelvic inflammatory disease (PID)	25 (4.7%)
Polycystic ovary syndrome (PCOS)	47 (8.8%)
Rheumatoid arthritis	9 (1.7%)
Scoliosis (curvature of the spine)	54 (10.1%)
Other spine problems	39 (7.3%)
Sjogren’s syndrome	2 (0.4%)
Lupus erythematosus	2 (0.4%)
Thyroid disease	32 (6.0%)
Ulcerative colitis	3 (0.6%)
Other chronic condition	228 (42.8%)
Have you been told that you were born with a structural problem/birth defect of your uterus, cervix, or vagina?	44 (8.9%)
General health and activities of daily living	N (%)
In general, would you say your health is good?	296 (55.5%)
Has there been a time in your life when you typically had pelvic pain during your periods?	522 (97.9%)
During your last period, did your pelvic pain prevent you from going to work or school or carrying out your daily activities (even if taking pain-killers)?	280 (70.9%)
During your last period, did you have to lie down for any part of the day or longer because of your pelvic pain?	356 (90.6%)
Does your health now limit you in bathing or dressing yourself?	134 (25.1%)
Does your health now limit you in lifting or carrying groceries?	253 (47.5%)
Does your health now limit you in moderate activities, such as moving a table, pushing a vaccum cleaner, bowling, or playing golf?	351 (65.9%)
Does your health now limit you in vigorous activities, such as running, lifting heavy objects, participating in strenuous sports?	477 (89.5%)
Does your health now limit you in walking one block?	138 (25.9%)
Does your health now limit you in walking several blocks?	272 (51.0%)
Does your health now limit you in walking more than a mile?	327 (61.4%)
Does your health now limit you in bending, kneeling or stooping?	295 (55.3%)
Does your health now limit you in climbing one flight of stairs?	169 (31.7%)
Does your health now limit you in climbing several flights of stairs?	355 (66.6%)

Note that not all Phendo participants completed the WERF survey.

### Unsupervised phenotype modeling

The proposed unsupervised mixed-membership method—fully described in the Methods section—models per-participant and per-question observations with a latent joint mixture of distributions, and outputs both groupings of responses that describe endometriosis phenotypes, as well as probabilistic assignments of each participant to the learned subtypes.

We evaluated the accuracy of the proposed model in describing unseen data (see results in Table 5), and observed a significant improvement of our method when compared to a vanilla mixed-membership baseline model—where responses to all questions are modeled together as in the topic model in^33^. We note the robustness of the learning process—there are no significant differences—with respect to specific choices of the hyperparameters of the model.

**Table 5:** 10-fold cross-validated test data log-likelihood of the proposed method Vs vanilla LDA.

Test data log-likelihood of the proposed method		
Model hyperparameters	Test log-likelihood for the model as in^33^ (mean ± std)	Test log-likelihood for the proposed model (mean ± std)
*K* = 2, *α* = 0.1, *β* = 0.1	−687951.86 (±47455.33)	−367855.70 (±27324.98)
*K* = 2, *α* = 0.1, *β* = 0.01	−688027.21 (±47424.77)	−367728.17 (±27179.73)
*K* = 2, *α* = 0.1, *β* = 0.001	−688049.86 (±47477.81)	−367781.96 (±27190.10)
*K* = 2, *α* = 0.01, *β* = 0.1	−689056.69 (±47443.14)	−368086.97 (±27197.90)
*K* = 2, *α* = 0.01, *β* = 0.01	−689270.81 (±47284.88)	−368368.67 (±27174.22)
*K* = 2, *α* = 0.01, *β* = 0.001	−689730.70 (±47553.67)	−368588.16 (±27689.93)
*K* = 2, *α* = 0.001, *β* = 0.1	−693364.63 (±47505.99)	−370060.29 (±27350.08)
*K* = 2, *α* = 0.001, *β* = 0.01	−693446.41 (±47418.04)	−369991.03 (±27165.18)
*K* = 2, *α* = 0.001, *β* = 0.001	−693645.14 (±47480.30)	−370052.10 (±27195.04)
*K* = 3, *α* = 0.1, *β* = 0.1	−681064.83 (±47169.90)	−364978.13 (±27062.30)
*K* = 3, *α* = 0.1, *β* = 0.01	−681003.93 (±47332.34)	−365462.25 (±27289.95)
*K* = 3, *α* = 0.1, *β* = 0.001	−681534.16 (±47021.27)	−365380.51 (±27122.67)
*K* = 3, *α* = 0.01, *β* = 0.1	−682631.13 (±47218.50)	−365766.74 (±26798.13)
*K* = 3, *α* = 0.01, *β* = 0.01	−682392.99 (±47130.02)	−365806.39 (±27076.40)
*K* = 3, *α* = 0.01, *β* = 0.001	−682620.18 (±47179.25)	−365807.88 (±27171.46)
*K* = 3, *α* = 0.001, *β* = 0.1	−686273.30 (±47539.28)	−367994.90 (±27034.03)
*K* = 3, *α* = 0.001, *β* = 0.01	−686666.58 (±47408.23)	−367859.95 (±26874.11)
*K* = 3, *α* = 0.001, *β* = 0.001	−686417.56 (±47156.44)	−367923.44 (±26942.59)
*K* = 4, *α* = 0.1, *β* = 0.1	−677435.77 (±47321.00)	−362748.07 (±26930.41)
*K* = 4, *α* = 0.1, *β* = 0.01	−677681.05 (±46751.86)	−363277.82 (±27292.59)
*K* = 4, *α* = 0.1, *β* = 0.001	−678124.44 (±46816.71)	−363310.62 (±26850.11)
*K* = 4, *α* = 0.01, *β* = 0.1	−678858.29 (±47393.52)	−364019.43 (±27006.19)
*K* = 4, *α* = 0.01, *β* = 0.01	−679569.77 (±47161.13)	−364008.15 (±27215.34)
*K* = 4, *α* = 0.01, *β* = 0.001	−679277.59 (±47150.13)	−364036.90 (±26933.38)
*K* = 4, *α* = 0.001, *β* = 0.1	−683839.21 (±46870.69)	−366149.54 (±26829.01)
*K* = 4, *α* = 0.001, *β* = 0.01	−683417.91 (±46932.56)	−366384.64 (±26828.00)
*K* = 4, *α* = 0.001, *β* = 0.001	−684045.97 (±47494.00)	−366304.03 (±27138.42)
*K* = 5, *α* = 0.1, *β* = 0.1	−674507.71 (±47127.03)	−361290.00 (±26836.91)
*K* = 5, *α* = 0.1, *β* = 0.01	−674681.24 (±47024.50)	−361318.58 (±26818.05)
*K* = 5, *α* = 0.1, *β* = 0.001	−675159.95 (±46797.63)	−361855.70 (±26851.60)
*K* = 5, *α* = 0.01, *β* = 0.1	−676658.40 (±47147.61)	−362468.01 (±27138.36)
*K* = 5, *α* = 0.01, *β* = 0.01	−676662.81 (±47356.85)	−362369.08 (±26737.76)
*K* = 5, *α* = 0.01, *β* = 0.001	−676309.70 (±46958.32)	−362585.89 (±27140.12)
*K* = 5, *α* = 0.001, *β* = 0.1	−681362.66 (±46825.38)	−364723.91 (±27100.00)
*K* = 5, *α* = 0.001, *β* = 0.01	−681469.84 (±47357.16)	−364799.82 (±27106.59)
*K* = 5, *α* = 0.001, *β* = 0.001	−681478.89 (±47564.73)	−364866.80 (±26866.35)

Notice the improvement in log-likelihood achieved by the proposed method when compared to the vanilla LDA model as in ref. ^33^.

The enigmatic nature of endometriosis and its poor clinical characterization makes indispensable the interpretability of the phenotyping model. The probabilistic posteriors learned by our model are highly interpretable and discriminative: the per-question posteriors describe how likely are participants within a phenotype to track specific responses. Due to the flexibility of our model in accommodating per-question modalities, the method is capable of capturing signal within each of the self-tracked variables separately, resulting in a better discrimination between endometriosis phenotypes. As such, our model selection is primarily guided by interpretability criteria.

In general, sparsity—using few per-question answers to describe each phenotype—helps experts understand the model outputs (i.e., the learned per-phenotype and per-question posterior distributions) better, as fewer answers become significant in discriminating among phenotypes. The selected model learned four phenotypes (as it captured distinguishing features, while models with more subtypes did not provide new discriminating insights) with sparse parameters (*α* = *β* = 0.001) that allowed endometriosis experts to easily interpret the provided outputs.

### Unsupervised phenotype modeling—Learned endometriosis phenotypes

**Fig. 2:**
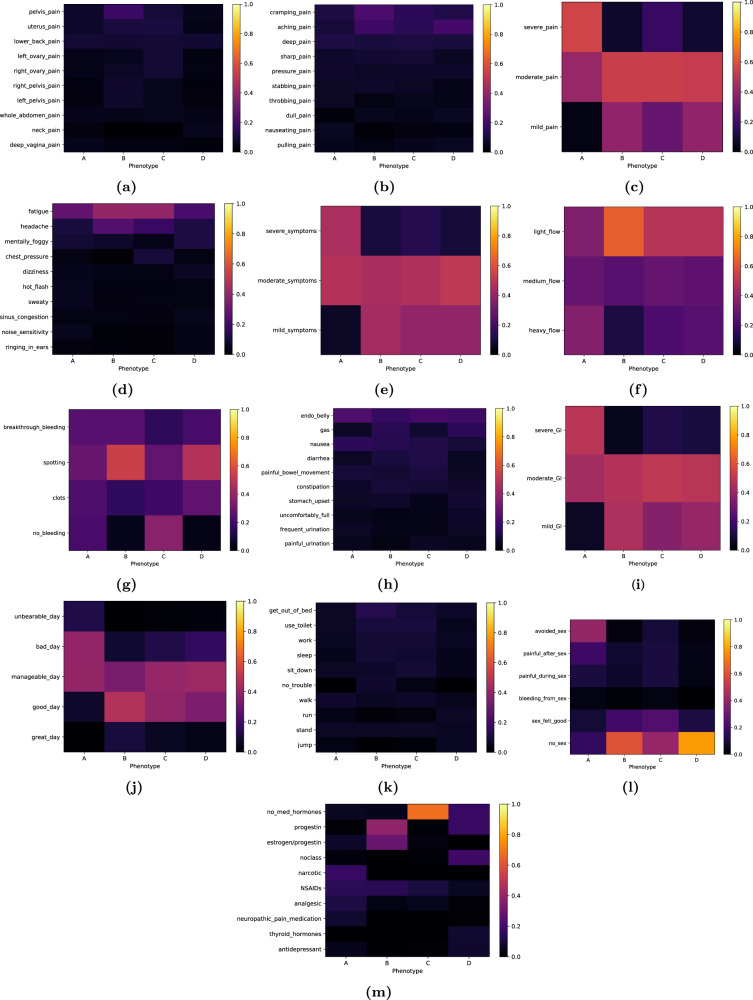
Visualization of learned posteriors for endometriosis phenotypes. Each phenotype is defined as a set of per-question probability distributions across the answers to each of the thirteen questions. Each heatmap represents the likelihood of the answers within a question for a given phenotype—for visual clarity, only the top 10 (most likely) vocabulary items of the posterior are displayed. **a** Where is the pain? **b** Describe the pain. **c** How severe is the pain? **d** What are you experiencing? **e** How severe is the symptom? **f** Describe your period flow. **g** What kind of bleeding. **h** Describe GI/GU system. **i** How severe is it? **j** How was your day? **k** Activities difficult to perform. **l** Describe sex. **m** Medications/hormones taken. For instance, the “*no_sex*” answer is highly likely to be tracked under phenotype D, and not likely to be tracked under phenotype A—yellow versus purple respectively, in heatmap **l**.

**Fig. 3:**
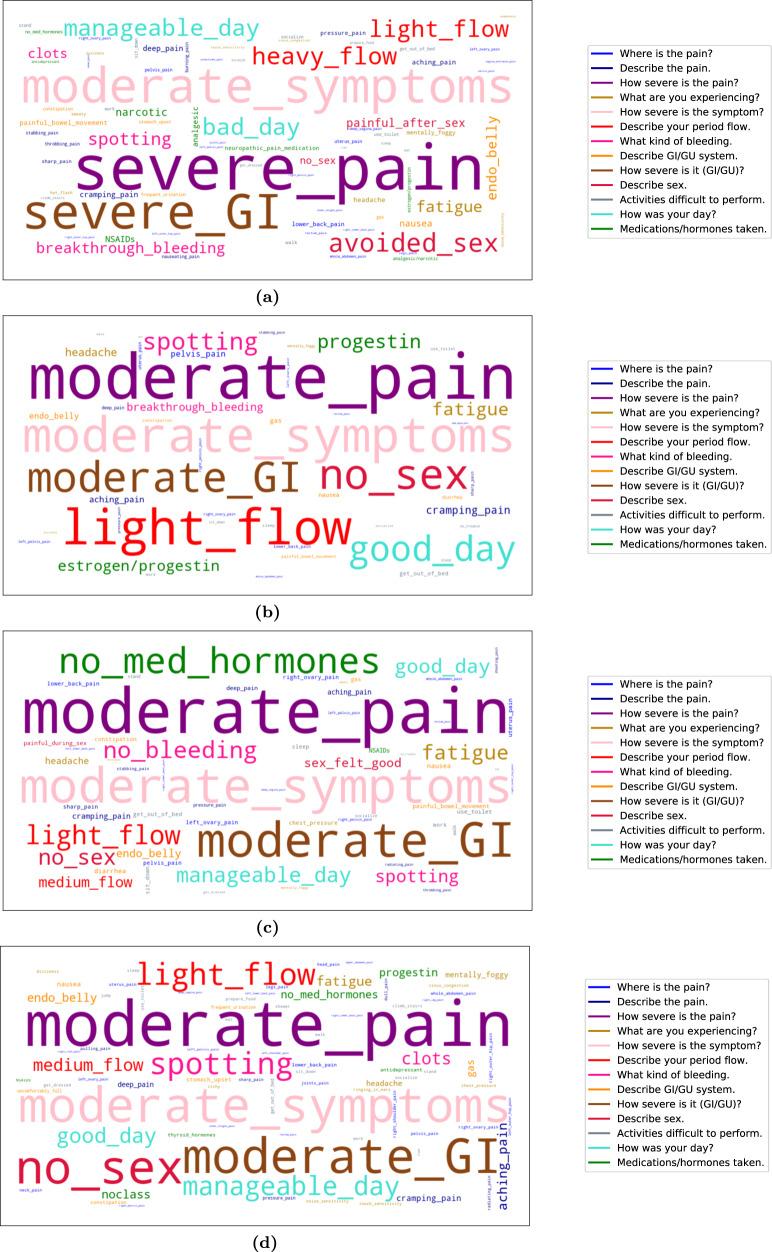
Answer-cloud visualization of learned endometriosis phenotypes. **a** Answer-cloud for phenotype A. **b** Answer-cloud for phenotype B. **c** Answer-cloud for phenotype C. **d** Answer-cloud for phenotype D. The font size of each answer reflects its likelihood to be tracked within the phenotype. Answers to the same question are depicted with the same color (see legend): e.g., “*no_sex*” and “*avoided_sex*”, shown in red, are two of the six potential answers to the sexual activity questions.

We present a summary of the outputs of the learned model for the whole study cohort in Figs. 2 and 3. The first illustrates the per-question posterior distribution for each phenotype, where for visual clarity, only the top 10 (most likely) vocabulary items of the posterior are displayed (the full vocabulary per-question posteriors are provided in the Supplementary Results). The second is an answer-cloud summary visualization of each phenotype (the per-question and per-phenotype answer-clouds are provided in the Supplementary Results). These figures reflect not only which responses are more commonly reported per phenotype (i.e., how likely is a participant within each subtype to track any of the per-question symptoms), but also how they correlate with each other in the Phendo cohort.

We report the following two main findings from the learned endometriosis phenotypes. First, each of the four phenotypes is uniquely characterized by distinct signs and symptoms, behaviors, and treatment strategies. Second, the learned phenotypes characterize endometriosis according to its severity—consistently across all signs and symptoms (pain, GI/GU, other symptoms)—and the burden on participants’ daily lives, hinting at the systemic aspect of the disease.

Phenotype A, specifically, describes a particularly severe endometriosis subtype. Furthermore, while the learned phenotypes reflect the state-of-knowledge about endometriosis, they highlight new insights and correlations across signs, symptoms, and treatments. We provide a detailed description of each phenotype per question, i.e., the posteriors in Fig. 2.

Across all learned endometriosis subtypes, chronic pain-related symptoms are common. However, there is a significant difference for phenotype A, as it is the only phenotype with significant posterior mass for “*severe pain*” (see Fig. 2c). The severity of other reported symptoms, such as gastrointestinal, genitourinary, and other symptoms, is also highest for phenotype A (Fig. 2i, 2e illustrate this, respectively).

For all participants in the cohort, the most salient pain locations tracked are pelvic, lower back, ovary and uterus—see overall answer-clouds in Fig. 3 and per-question visualizations in the Supplementary Results. A wider and more specific range of pain locations are likely to be reported by participants in phenotype A: there is significant evidence of deep vagina, vagina entrance and inner thigh pain, as well as cervix, rectum and intestine pain. On the contrary, phenotypes B and C are associated with pelvis, uterus or vagina pain primarily, while phenotype D has a less prominent, but broader association with pain locations. The tracked pain is commonly described as aching or cramping across all phenotypes, while phenotype A has higher likelihood of deep pain reports, and is uniquely likely to report burning, throbbing and nauseating pain.

Phenotypes learned by the model capture common endometriosis GI/GU symptoms of bloated abdomen (i.e., “*endo belly*”), as well as reports of constipation, diarrhea, and nausea. Phenotype A is more likely to report both nausea and irritable-bowel-like symptoms—congruent with the high prevalence of such syndromes in the disease—as well as to do so with higher severity. Phenotype A shows urinary-related symptoms as well.

Tracking of other symptoms of endometriosis (collected via the question “*What else are you experiencing?*” in Phendo) demonstrates the overall chronic nature of the disease. Fatigue, headache, mental fogginess, and dizziness are tracked across all learned phenotypes. Phenotype A uniquely experiences more systemic symptoms, like hot flashes, sweaty, and numbness; while phenotypes C and D are characterized by some symptoms of the upper abdomen, like chest pressure. Both phenotype A and D are likely to track noise- and touch-sensitivity, as well as sinus congestion.

In Fig. 2f, 2g, we observe that phenotypes B, C and D are likely to track light menstrual flow (with some evidence for medium flow as well), with spotting bleeding outside the period reported more significantly in phenotypes B and D. Phenotype A shows evidence of very irregular menstruation, and is the only subtype with heavy flow reports. Subtype A has higher likelihood of menorrhagia and clots, which appear less likely in phenotypes B and D.

Across all learned phenotypes, we observe a wide range of issues with daily activities, such as walking, standing, getting out of bed, using the toilet, sitting down, getting dressed, socializing, and working. Notice how salient these difficulties are for phenotype A, with basic functionalities like walking, standing or getting out of bed being commonly reported.

In general, phenotype A experiences low quality of life with high probability. Specifically, subtype A is uniquely associated with “*bad*” days—see high posterior mass in Fig. 2j—while the rest of the phenotypes are likely to track on the other side of the spectrum: i.e., “*manageable*” and “*good*” days. This effect is also evident with regards to sex, as phenotype A is the only subtype where sex is explicitly avoided, or reported to be painful (see Fig. 2l).

Finally, we observe that medications and hormones are highly discriminative of how different patients experience endometriosis. From the learned phenotypic posteriors (see Fig. 2m), we conclude that phenotype A is uniquely associated with the use of narcotics and neuropathic pain medications, phenotype B with hormonal treatments, phenotype C with no medical treatments, and phenotype D with a wider variety of treatments (hormonal, narcotic and antidepressants).

### Unsupervised phenotype modeling—learned participant phenotypic assignments

**Fig. 4:**
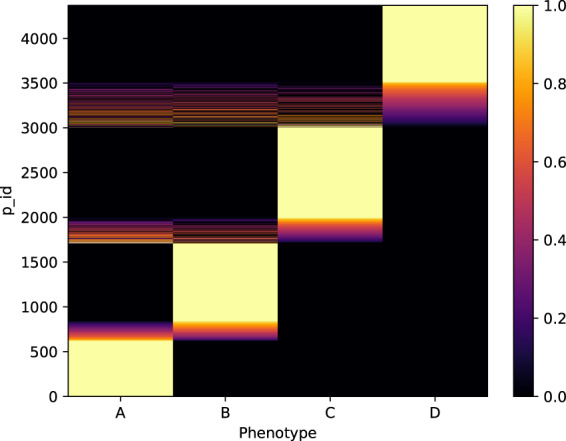
Posterior assignment probability of each participant across the phenotypes learned by the model. While the model provides membership probabilities for each participant across phenotypes, most participants are clearly assigned to a single phenotype (assignment probability above 0.9, in yellow in the heatmap).

Fig. 4 provides the probabilistic assignment of participants to the learned phenotypes. While the model provides for each participant membership probabilities across all phenotypes, we see that most participants are clearly assigned (with probability above 0.9) to a single phenotype.

One possible question when learning unsupervised clustering of participants is whether the self-tracking patterns of the participants is responsible for their underlying phenotype assignments or, rather, whether their assignments are uncovering actual endometriosis characteristics. In our data, we note that the average number of days tracked in all learned phenotypes are similar (34, 48, 41, and 27 on average), although participants associated with phenotype A tracked slightly more observations (on average, 116, 80, 80, and 66, respectively).

**Fig. 5:**
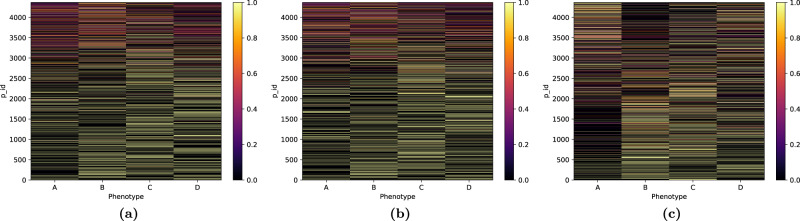
Learned phenotype assignments are not correlated with the number of days, number of observations tracked, nor the ratio of observations per day tracked by participants. Posterior assignment probability of each participant across the phenotypes learned by the model, ordered by **a** number of days, **b** number of observations, and **c** ratio of observations per day tracked by each participant. We observe no correlation between the phenotype assignments and the number of days, number of observations tracked, nor their ratio.

In contrast, the phenotypic assignments of participants do not correlate with the number of days or the observations participants tracked, nor their ratio (see Fig. 5). The learned phenotypes do not capture spurious self-tracking patterns related to engagement with the app, but rather represent participants based on their answers to endometriosis relevant Phendo questions.

### Endometriosis phenotype evaluation

On top of the checks presented in the previous section related to the coherent representation of the learned phenotypes, as well as to a meaningful clustering of different types of endometriosis patients, we further assess the quality of the learned phenotypes in two ways: how they correlate with expert endometriosis groupings, and how they associate with responses to the WERF survey.

### Phenotype evaluation—agreement between expert clustering and phenotyping

The responses collected by the Phendo app of randomly selected 40 participants were reviewed by two endometriosis experts, who were asked to group them based on their clinical understanding of patient signs and symptoms (see guidelines description in the Methods section). In general, experts tended to categorize participants based on the symptomatic intensity (mild Vs severe) and the clinical management of the disease (no medical involvement Vs clinically managed).

The assignments by the experts and the model are compared, via confusion matrices (provided in Tables 6 and 7). High cluster purity values were attained for both the severe phenotype A (0.9 and 0.8) and the mildest phenotype B (0.775 and 0.7)—see Tables 8 and 9—indicating a clear agreement between our model and the experts on which participants were assigned to the two ends of the endometriosis spectrum (the inter-expert purity is 0.85 and 0.75 for the severe and mild cases, respectively).

**Table 6:** Phenotype confusion matrix for Expert 1.

Phenotype confusion matrix for Expert 1				
	Expert 1	Expert 1	Expert 1	Expert 1
	Phenotype A	Phenotype B	Phenotype C	Phenotype D
Model Phenotype A	7	0	0	2
Model Phenotype B	1	6	3	1
Model Phenotype C	0	3	5	2
Model Phenotype D	1	1	2	6

**Table 7:** Phenotype confusion matrix for Expert 2.

Phenotype confusion matrix for Expert 2				
	Expert 2	Expert 2	Expert 2	Expert 2
	Phenotype A	Phenotype B	Phenotype C	Phenotype D
Model Phenotype A	5	2	1	1
Model Phenotype B	2	9	0	0
Model Phenotype C	1	5	1	3
Model Phenotype D	1	3	3	3

**Table 8:** Confusion matrices for severe cases.

Confusion matrices for severe cases				
	Expert 1	Expert 1	Expert 2	Expert 2
	Severe case	Non-severe case	Severe case	Non-severe case
Model Severe case	7	2	5	4
Model Non-severe case	2	29	4	27

**Table 9:** Confusion matrices for mild cases.

Confusion matrices for mild cases				
	Expert 1	Expert 1	Expert 2	Expert 2
	Mild case	Non-mild case	Mild case	Non-mild case
Model Mild case	6	5	9	2
Model Non-mild case	4	25	10	19

The cluster purity for the full phenotypic assignments learned by the model is lower (0.6 and 0.55), reflecting the hard time experts had splitting some participants into 2 subtypes within the moderate group. We noticed that, for some of the participants for which the experts had assignment uncertainty, there were few self-tracked variables (both in quantity and in clinical relevance). Besides, after revealing the model assignments to the experts, they noticed how the model was distinguishing between moderate phenotypes based on certain variables that were non-critical in state-of-the-art recommendations, such as treatment choices, menstruation flow and sex-reports, which they had not previously considered.

### Phenotype evaluation—associations between learned phenotypes and survey answers

To further validate the insights from the proposed unsupervised model, we study the statistical association between the learned phenotypes and the participant responses to the WERF survey. In general, the severity and quality of life indicators of endometriosis (as specified by WERF standards) align well with how our model discriminates patients. Specifically, the most significant associations occur for daily living limitations, the surgical burden associated with the disease, and their overall health.

Quality of life is considerably impacted for participants assigned to phenotype A: they are significantly more likely to rate their overall health as poor in their WERF-EPHect responses, with those in phenotypes B and C being associated with good or excellent self-evaluations. More precisely, those in phenotype A are distinctively associated with responses acknowledging limitations on activities like bending, kneeling, stooping, lifting or carrying groceries, bathing, dressing, walking or climbing stairs. They are also associated with limitations for running, lifting heavy objects or participating in other strenuous sports. Participants assigned to both phenotypes A and D have reported significant pelvic pain preventing them from going to work or school, as well as from carrying out other daily activities.

The severity of endometriosis for participants in phenotype A is evident when looking at the surgical burden as well: they are more likely to have undergo abdominal surgeries (e.g., gallbladder surgery), and are associated with more surgical procedures for endometriosis (average of 2.32 for phenotype A, versus 1.62, 1.51, 1.46, respectively for other phenotypes), as well as laparoscopies (1.76 versus 1.40, 1.40, and 1.26 respectively). It is interesting to observe that phenotype A and D are both associated with evidence of fibromyalgia and sigmoidoscopy or colonoscopy procedures. Hormone-induced menstruation is uniquely associated with phenotype B, while participants assigned to phenotype C are the only ones associated with regular periods.

We found that participants assigned to phenotype A are most likely to have pelvic inflammatory diseases, with some evidence of high blood pressure associated with phenotypes A and C. Migraine is associated with phenotype A, while chronic fatigue syndrome and anxiety disorders requiring medication or therapy were associated with both participants in phenotypes A and D. In general, even if several comorbidities such as PCOS or interstitial cystitis are high in the overall cohort (see Table 4), no significant association was found with any particular learned subtype.

We conclude by noting that we find a weak association between participants assigned to phenotype A and higher body mass index (BMI), while no significant correlations are found between phenotypes and age, race, time to diagnoses, or reports of diagnosis of endometriosis within the family.

## Discussion

Our joint modeling of multiple self-tracked variables through mixed-membership models show that we can produce robust, clinically meaningful groupings of self-tracked signs and symptoms collected via patient-centered mobile and digital platforms.

We find that the proposed unsupervised method learns robust phenotypes, with respect to specific choices of the hyperparameters of the model and the randomness associated with inference. We observe that the log-likelihood of the selected model is stable for different realizations of the inference algorithm, as well as to different train/test splits. Overall, the learned phenotypes show the same discriminative features, and the set of significant associations between the participant phenotypic assignments and the WERF questionnaire responses are consistent across realizations.

Even if the available data is heterogeneous, both in type and quantity across participants, the proposed method is robust to the inherent uncertainties of self-tracked data, and does not pick up spurious signals—the learned phenotypes do not correlate with the number of observations or days tracked, nor other variables like age or race of participants.

The proposed model characterizes the burden of endometriosis across all the learned phenotypes. The learned (unsupervised) subtypes, along with participant phenotypic assignments, align well with previous clinical knowledge about endometriosis, but also suggest novel findings. Our approach reflects direct patient experiences with endometriosis, and provide potentially novel insights about the disease.

The reports from the WERF survey confirm that patients with endometriosis have a higher number of known comorbidities than the general US population (see Table 4). These include autoimmune, endocrine-based, and mental health disorders, such as irritable-bowel syndrome^34^, Hashimoto’s disease^35^, fibromyalgia^36^, anxiety disorders^37^, asthma^38^, chronic fatigue syndrome^39^, depression^40^, migraine^41^, and PCOS^42^.

The clusters of symptoms learned for the different phenotypes confirm, as well, the chronic nature of endometriosis: fatigue, headaches, mental fogginess, gastrointestinal problems, and pain reports are common across all phenotypes. These symptoms (specially fatigue and mental fogginess or dizziness) are similar to those experienced in other complex chronic conditions, and are characteristic of low grade inflammation^43^.

The observed commonality of pelvic and lower back pain symptoms across phenotypes is expected for endometriosis patients^44^, as well as having gastrointestinal symptoms related to irritable-bowel syndrome^45,46^. Our analysis shows spotting and bleeding outside of the period to be characteristic of all participants in our cohort, which matches findings connecting premenstrual spotting with histologically confirmed endometriosis^47^.

The phenotypes learned by the proposed model separate participants’ experiences according to their severity, consistently across all signs and symptoms (pain, GI/GU, other symptoms). Specifically, Phenotype A describes a particularly severe endometriosis subtype.

First, we observe (both in the learned posteriors and in the computed associations) that patients assigned to subtype A track symptoms related to several comorbidities already reported in the literature. Diagnosis of endometriosis has been linked to anxiety, depression, and other mood disorders^48,49^, migraines^50^, high blood pressure^51^, PCOS^52^, and chronic fatigue syndrome^6,53,54^. The significant associations found for phenotype A reflected a higher surgical burden, and a lack of adequate treatment of the disease. This finding is consistent with the existing literature studying endometriosis diagnosis^55–57^.

The severe genitourinary symptoms characteristic of phenotype A (e.g., painful urination or dysuria) have been previously reported in the literature^58–60^, but their association with the collection of other symptoms tracked within this phenotype is novel. Associations with the WERF survey were consistent with current knowledge regarding menstruation, but also demonstrated novel patterns of the disease. Specifically, menstrual irregularity has been shown to be associated with endometriosis before, but not with a specific subgroup of participants^61,62^. Phenotype A shows a higher likelihood of disordered periods (with heavier flows and menorrhagia). Besides, participants assigned to this subtype have tracked menstrual bleeding, and are associated with irregular periods in their WERF survey responses as well—only participants assigned to phenotype C were associated with regular periods. Even if menorrhagia is a common endometriosis symptom^63^, it has not been previously associated with a particular subgroup of endometriosis patients. Furthermore, hormone-induced menstruation is uniquely associated with phenotype B, which aligns well with the presence of hormonal treatments found in the medication posterior of Fig. 2m.

Painful sex is a widely known symptom for endometriosis^64–66^. We here find dyspareunia to distinctively correlate with phenotype A. This finding is consistent with the highly systemic nature of the disease, the impact of gastrointestinal and genitourinary symptoms, and pain locations—intestines, cervix pain, vagina entrance pain—specifically highlighted by the posteriors learned for phenotype A. The literature has previously documented sexual problems and active avoidance of sexual activity by women with endometriosis^67,68^. However, we here find a novel association between dyspareunia and a specific subtype of the disease.

The learned phenotypes provide evidence of the different treatment alternatives for the disease, each endometriosis subtype being characterized by distinct medication intakes. A first line of treatment for endometriosis symptoms is often a combination of progestin and/or hormonal medications^69^, which interestingly are highly associated with learned phenotype B, while phenotype C is not correlated with any particular medication. On the contrary, phenotype A is characterized by a heavy use of narcotics, and a more likely use of antidepressants and neuropathic pain medications (with some evidence of this also appearing in phenotype D). This finding reflects the psychological and physiological impact of the disease, as neuropathic pain often develops when there is damage to the somatosensory nervous system: evidence suggests that women with endometriosis, and in particular those with pain in the upper anterior-lateral part of the thigh (which is uniquely represented in pain locations for phenotype A), tend to experience neuropathic pain^70^.

The impact of the disease on the quality of life aligns with the severity of symptoms across the learned phenotypes. Problems with day-to-day functioning of endometriosis patients have been previously documented^71^, and the associated loss of productivity and reduced quality of life is well known in the literature. However, evaluating the differences among patient subgroups is yet unexplored^71–73^. Here, we find that “*Bad days*” and “*Poor health*” reports—in the Phendo app, as well as in the WERF survey—are uniquely associated with phenotype A, while participants in other phenotypes don’t report such negative experiences. The impact of the disease on quality of life and daily activities is supported by both the learned phenotype posteriors and the responses to the WERF survey. There is a clear and significant association between problems with daily living activities and participants assigned to phenotype A.

The exact etiology of endometriosis remains unclear^74^. Among studies that examined heritability of the disease, there seems to be both maternal and paternal genes involved in the development of endometriosis, but the majority appear paternally inherited^75^. In our study, 38% of participants reported a diagnosis of endometriosis within the family, but no significant etiology association was found at the phenotype level. Underweight BMI has traditionally been thought of as a risk factor for endometriosis, but recent research suggests that among woman who are obese, the disease is more severe^76^. Our analysis points to a weak association between BMI and a more severe experience of the disease.

Finally, we also found some reports of tinnitus—ringing in ears—and itchiness (mostly for phenotype D), which have not been documented as important symptoms for endometriosis in the literature. Participants associated with this phenotype may be impacted by changes in hormone levels, which at least for menopausal women, have been associated with tinnitus^77^ and itchiness^78^.

As a first step towards investigating phenotyping of endometriosis based on self-tracked data, this study has ignored the temporal aspect of the condition, and have instead aggregated all tracked observations for each participant. We acknowledge that the heterogeneity in tracking might vary within a given participant’s timeline as well. Even if it is plausible that there is signal across learned phenotypes and disease progression, there is a lack of medical evidence as to whether endometriosis phenotypes indicate progression of disease. Specifically, there is little evidence that superficial endometriosis progresses to deep endometriosis^8^. Furthermore, our analysis shows no correlation between the discovered phenotypes and age or time to diagnosis.

Future work should consider modeling the temporality of the signs and symptoms of endometriosis, particularly since it is estrogen dependent and linked to the menstrual cycle. We acknowledge that how robust the learned phenotypes are when compared to other advanced computational phenotyping techniques, such as^79^, is an open research question. We also note that our association analysis may be limited, both in terms of the type of questions available in the WERF survey, and the number of participants for whom we were able to collect responses.

Nevertheless, we argue that the analysis in this study already sheds novel insights into the understanding of endometriosis subtypes, and demonstrates the value of patient-generated data and unsupervised learning methods in medical research. This paper contributes to research in digital phenotyping from self-tracking data, and highlights how patient-powered mobile and digital technologies can be leveraged, in combination with unsupervised machine learning techniques, to study diseases and health outcomes.

In the case of endometriosis, a particularly enigmatic condition with a dire need for phenotyping, our method identified four subtypes of patients, grouped by severity of their condition and other factors of interest. Moreover, clinically meaningful novel associations beyond what is currently known about the disease were identified.

## Methods

### Unsupervised phenotyping model

We aim at understanding how self-tracked data from smartphones—a set of heterogeneous signs and symptoms from an enigmatic disease—can be grouped into different phenotypic experiences. Self-tracking data raises several considerations—it is irregularly sampled, noisy and contains several different data types—that we need to account for.

The process of extracting clinically relevant characteristics from a collection of data is generally defined as computational phenotyping. One family of phenotyping approaches are the generalized low-rank models (GLRMs), where the clinical data is put into a matrix form *A*, and a low-rank decomposition into factors *X* and *Y* is searched for^80^. The factor *X* represents each observation in *A* in terms of low-rank features *Y*, which encodes a low-rank feature representation of the original data. This factorization is found via an optimization procedure that consists of a loss function and corresponding regularizing terms. Particular choices of loss and regularization functions result in many well-known models. For instance, a mean squared-loss and no regularization is mathematically equivalent to principal components analysis (PCA). After finding a good low-rank representation, clustering techniques (such as K-means) are applied in their latent feature representation to derive cluster centroids (i.e., phenotypes are vectors in the embedded space). We provide a description of GLRM baselines and their performance in the Supplementary Methods, which did not discover clinically meaningful endometriosis phenotypes.

The goal of these GLRMs^80^ and other methods, such as non-negative tensor factorization^81^, is to autonomously identify clusters, usually in the learned latent space. Even if progress has been made on learning sparse and diverse phenotypes^79^, interpretation of the learned clusters to clinicians is challenging. In general, a cluster centroid vector in latent space lacks clinical meaning, while the explanation of the centroid in the original space demands a complicated understanding and explanation of a high-dimensional vector of clinical features. Besides, when using non-linear embedding functions, the mapping from latent to original features becomes even more convoluted.

In this work, we leverage an unsupervised probabilistic method to account for the lack of gold-standard labels (i.e., supervised methods are not applicable), and the heterogeneity of the symptomatic experience (i.e., we aim at a probabilistic assignment of shared signs and symptoms across patients). We propose an extended mixed-membership model^82,83^, which is a Bayesian generative model that can accommodate the inherent heterogeneity and uncertainty of the data, to capture the latent structure of collections of groups of self-tracked signs and symptoms.

Topic models^84^ are one of the primary examples of mixed-membership models, where one infers the latent topics of a corpora of documents. Intuitively, if a document is about a particular topic, one would expect specific words to appear in the document more or less frequently. However, a document typically covers multiple topics in different proportions. Topic models capture this intuition mathematically, based on the statistics of the observed words in each document, and outputs what the topics might be, as well as the document’s proportion of topics^33^.

Here, we cast the set of self-tracked responses per participant as “documents”, all generated from the “corpus” of endometriosis patients. As such, each set of tracked observations is modeled as a mixture model, where the mixture components (i.e., the phenotypes) are shared across the population, but the mixture proportions vary per participant.

The available self-tracked data however is not a standard document, but a collection of responses to different questions—for the unsupervised learning of phenotypes, we only use the self-tracked data, not the WERF EPHect questionnaire data, which is left-out for evaluation purposes. The Phendo app already provides a fixed set of possible responses to most of the questions, and medications and hormones were mapped to their corresponding medication classes of a fixed size (see per-question vocabularies in the Supplementary Results).

As a competitive baseline for the task at hand, we consider the mixed-membership model known as Latent Dirichlet Allocation^33^. For this approach, the collection of responses to different questions *q* = {1, ⋯, *Q*} are concatenated. The input to this baseline is a high-dimensional (*V*_1_ + *V*_2_ + ⋯ + *V*_*Q*_) multinomial vector per participant, where *V*_*q*_ is the vocabulary size of each question *q*, which the method uses to learn “topics” (i.e., phenotypes) and the per-participant assignments to each phenotype.

We here extend as in ref. ^83^ the mixed-membership model to accommodate for multi-modal data, where each modality is an specific question *q* = {1, ⋯, *Q*} with its vocabulary size *V*_*q*_. The proposed mixed-membership model infers phenotypes based on the co-occurrence of observations across the set of per-question responses and participants. The probabilistic graphical model and full details of the relevant statistical functions are provided in the Supplementary Methods and ref. ^83^. The proposed unsupervised method outputs groupings of per-question responses to self-tracked variables that describe endometriosis phenotypes. The learned probabilistic posteriors per-question (see Fig. 2) describe how likely are certain terms to be tracked for each phenotypic profile.

In order to determine the hyperparameters for the task at hand, we perform held-out data log-likelihood comparisons (10-fold cross-validation), where the data are split with a 80/20 train/test ratio, the hyperparameters are varied within *K* ∈ {2, 3, 4, 5}, *α* ∈ {0.1, 0.01, 0.001}, and *β* ∈ {0.1, 0.01, 0.001}. Since computing the log-likelihood of mixed-membership models for unseen data is nontrivial—see discussion in ref. ^85^—we extend the “left-to-right” method proposed in ref. ^85^ to our per-question mixed-membership model.

### Phenotype visualization

To allow for easy and visually appealing clinical evaluation, we provide posterior heatmaps, and a visual summary of each phenotype’s most prominent responses via answer-clouds (see Figs. 2 and 3, respectively). The former allows for a clear identification of the most salient responses, as they show the most discriminative vocabulary items per-question. Answer-clouds (also known as tag-clouds or word-clouds) are a novelty visual representation of text data. Shown answers are single vocabulary items per-question in the Phendo app (full list of answers are provided in the first section of the Supplementary Results), where the color indicates the question type, and the font size reflects the importance of each item in the learned phenotype. This format is commonly used for quickly presenting the most prominent terms to determine its relative prominence in the data. Due to the different vocabulary sizes for each considered Phendo question, comparing posteriors with different support is challenging. In this work, the answer-clouds are plotted by conditioning on the vocabulary items that cover 80% of the posterior mass per-question. As such, the relative size of visualized responses match the proportions of the conditional probability ratios. This allows for a more clear identification of the most salient responses per-question, even with different sized vocabularies per-question.

### Agreement between expert clustering and unsupervised phenotyping

We randomly selected 40 participants from the cohort, who had at least 30 days of activity with more than 100 tracked observations, for the experts to review. We selected 8 participants per phenotype that had high posterior probability (above 95% percent) of being assigned to a unique phenotype, and 8 additional participants for which the model output was uncertain (where at least 80% of the probability of phenotype assignment was shared by more than one subtype). The participant responses collected by the Phendo app were reviewed by two endometriosis experts, who were asked to group them based on their clinical understanding of patient signs and symptoms. The guidelines for the experts to review were written separately from the execution of the proposed unsupervised modeling algorithm. Specifically, endometriosis experts where instructed to categorize participants into groups according to their clinical understanding of patient signs and symptoms, i.e., following their endometriosis knowledge and expertise. As a secondary task, they were asked to provide an explanation of how they used the available data (i.e., the self-tracked responses to the Phendo questions, which are different from state-of-the-art clinical data) to group the participants, and how such data supported their understanding of the disease. The assignments by the experts and the model are compared via confusion matrices.

### Associations

We compute statistical associations between phenotypes learned by the model and responses to the questions from the WERF EPHect questionnaire^32^. After learning the model, participants were assigned to phenotypes based on the maximum per-phenotype posterior probability, and associations computed between responses to the WERF responses of participants within each subtype. For categorical questions, the chi-square test of independence of variables in the contingency table per phenotype was computed^86^. For questions with continuous outcomes, the Kruskal—Wallis H-test for independent samples per phenotype was computed^87^. This is a non-parametric version of ANOVA that works on 2 or more independent samples, which may have different sizes, and tests the null hypothesis that the population median of all of the groups are equal. We report correlations at a significance level of 0.05.

### Ethics

Data collection and the analysis presented in this work were carried out under Research Protocol #AAAQ9812 approved by Columbia University IRB. We obtained signed informed consent from all participants in the study.

### Reporting summary

Further information on research design is available in the Nature Research Reporting Summary linked to this article.

## Supplementary information

Supplementary Information

Reporting Summary

## Data Availability

Please contact the authors to obtain access to a de-identified version of the data that supports the findings of this study through a data-use agreement.

## Code availability

Our code has been developed using open source tools in Python with common statistical libraries (e.g., NumPy, SciPy and Pandas). The code required for processing the data and producing the presented results is available in the public GitHub repository https://github.com/iurteaga/phendo.
