# Supplementary material for: Learning endometriosis phenotypes from patient-generated data
Source: NPJ Digit Med. 2020 Jun 24;3:88. doi: 10.1038/s41746-020-0292-9 (PMC7314826; doi:10.1038/s41746-020-0292-9)
Supplement: Supplementary file 1 — Supplementary Information [file 41746_2020_292_MOESM1_ESM.pdf]

Learning endometriosis phenotypes from patient-generated data: Supplementary information

1 Supplementary Results

1.1 Per-question answer vocabulary

- **Where is the pain?**: bones_pain, cervix_pain, deep_vagina_pain, diaphragm_pain, head_pain, inner_thighs_pain, intestines_pain, joints_pain, left_arm_pain, left_breast_pain, left_leg_pain, left_lower_back_pain, left_outer_hip_pain, left_ovary_pain, left_pelvis_pain, left_ribs_pain, left_shoulder_pain, left_side_abdomen_pain, legs_pain, lower_back_pain, lower_chest_pain, neck_pain, pelvis_pain, rectum_pain, right_arm_pain, right_breast_pain, right_leg_pain, right_lower_back_pain, right_outer_hip_pain, right_ovary_pain, right_pelvis_pain, right_ribs_pain, right_shoulder_pain, right_side_abdomen_pain, upper_abdomen_pain, upper_chest_pain, uterus_pain, vagina_entrance_pain, whole_abdomen_pain
- **Describe the pain**: aching_pain, burning_pain, cramping_pain, deep_pain, dull_pain, nauseating_pain, pressure_pain, pulling_pain, pulsating_pain, radiating_pain, sharp_pain, shooting_pain, stabbing_pain, throbbing_pain, twisting_pain
- **How severe is the pain?**: mild_pain, moderate_pain, severe_pain
- **What are you experiencing?**: allergies, asthma, blurry_vision, chest_pressure, dizziness, eczema, fatigue, fever, headache, hives, hot_flash, itchy, mentally_foggy, noise_sensitivity, numbness, rash, ringing_in_ears, sinus_congestion, sweaty, swelling, touch_sensitivity
- **How severe is the symptom?**: mild_symptoms, moderate_symptoms, severe_symptoms
- **Describe your period flow**: heavy_flow, light_flow, medium_flow
- **What kind of bleeding**: breakthrough_bleeding, clots, no_bleeding, spotting
- **Describe GI/GU system**: blood_in_stool, cant_urinate, constipation, diarrhea, endo_belly, frequent_urination, gas, heartburn, mouth_sores, nausea, painful_bowel_movement, painful_urination, stomach_upset, uncomfortably_full, vomiting
- **How severe is it?**: mild_GI, moderate_GI, severe_GI
- **Describe sex**: avoided_sex, bleeding_from_sex, no_sex, painful_after_sex, painful_during_sex, sex_felt_good
- **Activities difficult to perform**: climb_stairs, eat, get_dressed, get_out_of_bed, have_sex, housework, jump, kneel, lie_down, lift, no_trouble, prepare_food, run, shop, shower, sit_down, sleep, socialize, stand, stretch, use_toilet, walk, work
- **How was your day?**: bad_day, good_day, great_day, manageable_day, unbearable_day
- **Medications/hormones taken**: adrenergic_agonists, amphetamine, analgesic, analgesic/narcotic, analgesic/nsaids, analgesic/opioids, anesthetic, anorectic, anti-inflammatory, antiacid, antiacid/nsaids, antibiotics, anticholinergic, anticoagulant, anticonvulsant, antidepressant, antidiabetic_medication, antidiarrheal, antiemetic, antihistamine, antihypertensive, antipsychotic, antispasmodic, antispasmodic/sedative, anxiolytic, anxiolytic/anesthetic/muscle_relaxant, barbituate, barbituate/analgesic, beta_blocker, bronchodilator, calcium_channel_blocker, cough_medicine, decongestant, diuretic,

dopamine_agonist, estrogen, estrogen/progestin, gonadotropin-releasing_hormone_agonist, gonadotropin-releasing_hormone_antagonist, hormone_based_chemotherapy, hormone_replacement_therapy, human_chorionic_gonadotropin, human_follicle_stimulating_hormone, laxative, muscle_relaxant, narcotic, narcotic/nsaids, neuropathic_pain_medication, no_med_hormones, noclass, nonbenzodiazepine_hypnotic, nsaids, opioids, progestin, sedative, statin, steroid, stimulant, thyroid_hormones, topical_anti-tumor_medication, triptan, vasoconstrictor, vitamin_a_derivative

1.2 Per-question posteriors

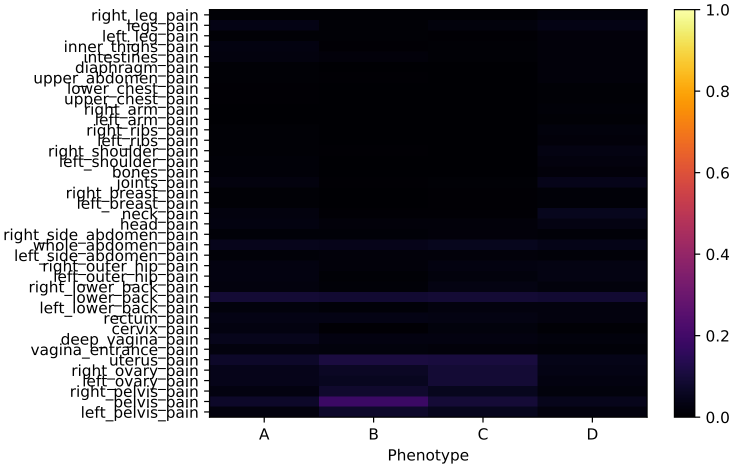

(a) Where is the pain?

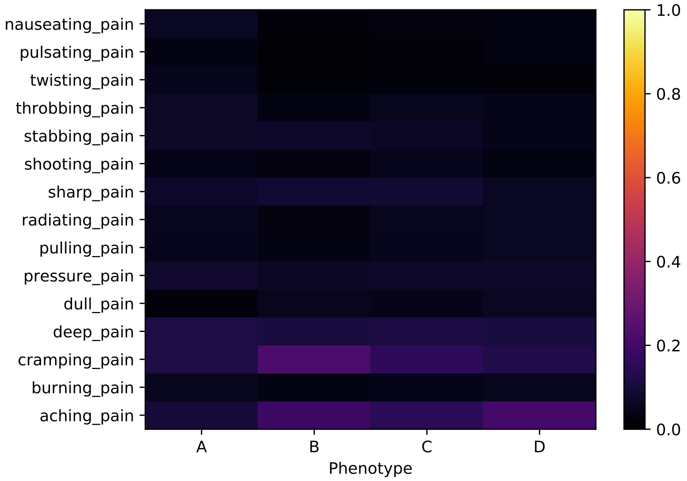

(b) Describe the pain.

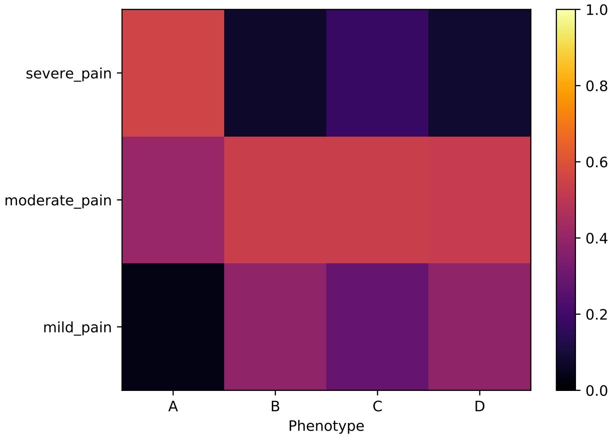

(c) How severe is the pain?

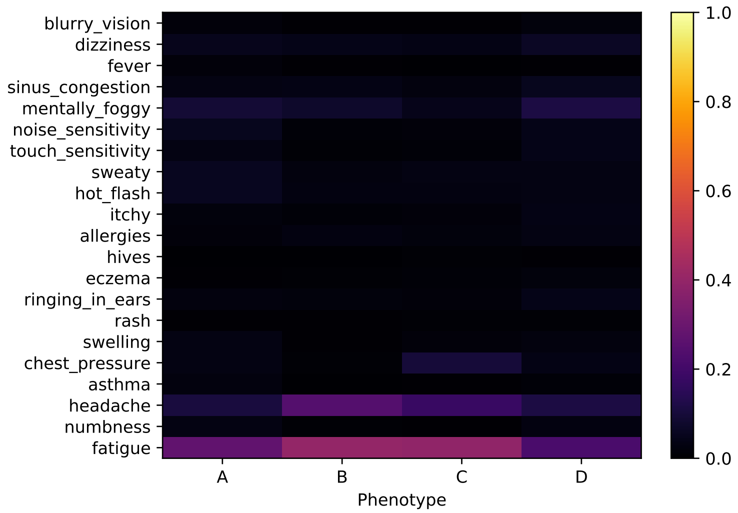

(d) What are you experiencing?

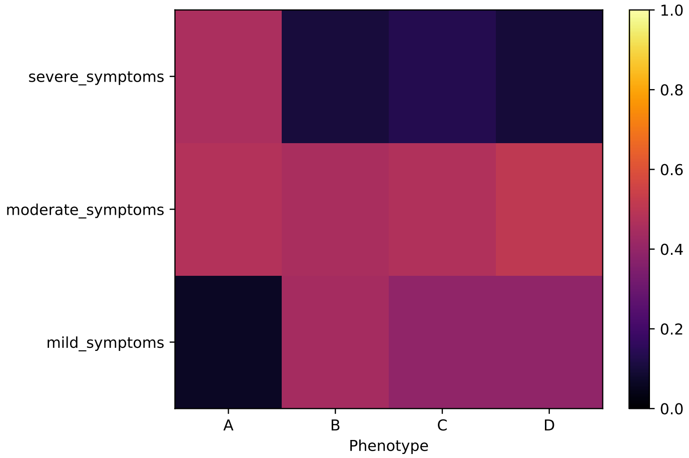

(e) How severe is the symptom?

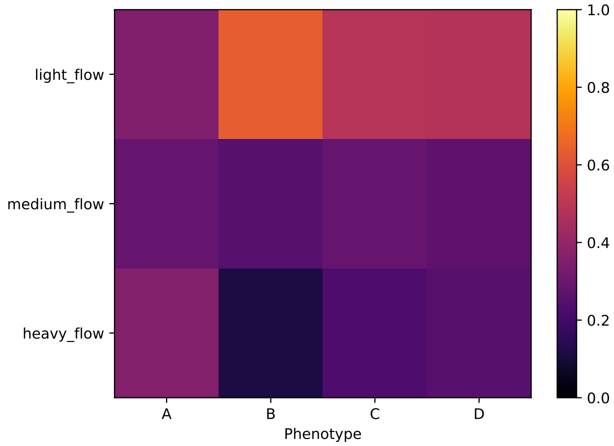

(f) Describe your period flow.

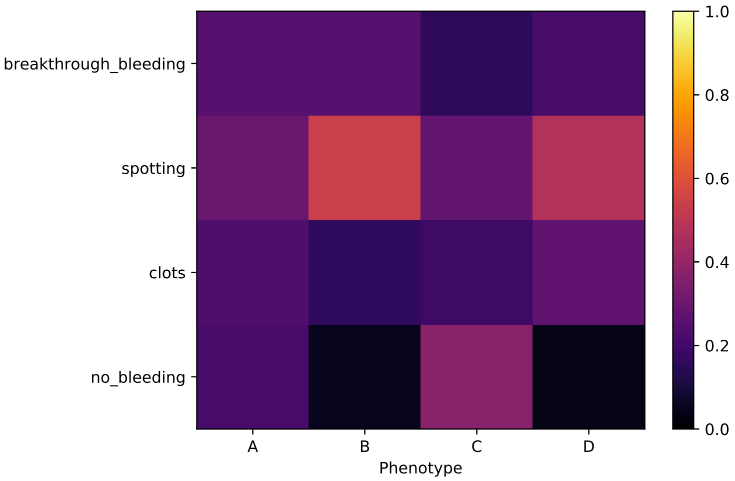

(g) What kind of bleeding.

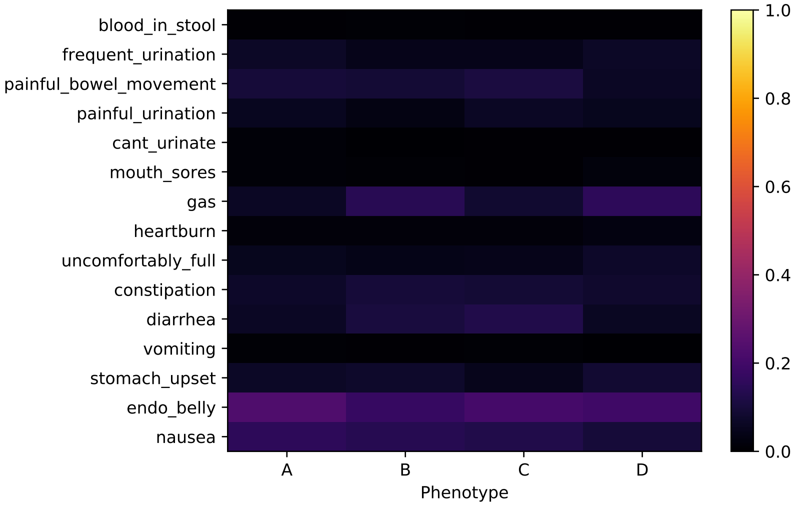

(h) Describe GI/GU system.

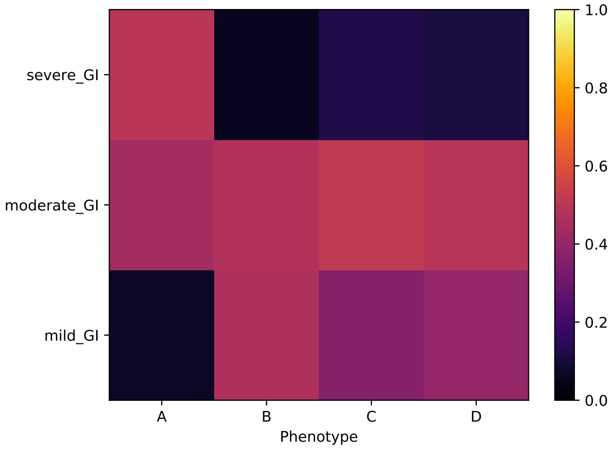

(i) How severe is it?

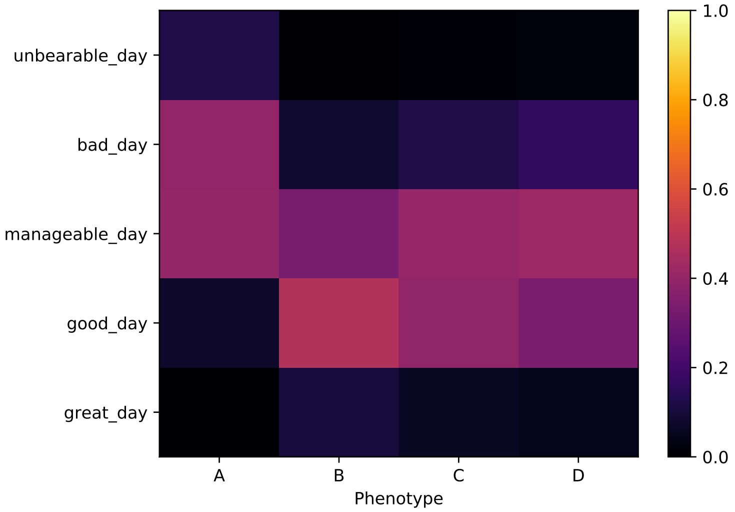

(j) How was your day?

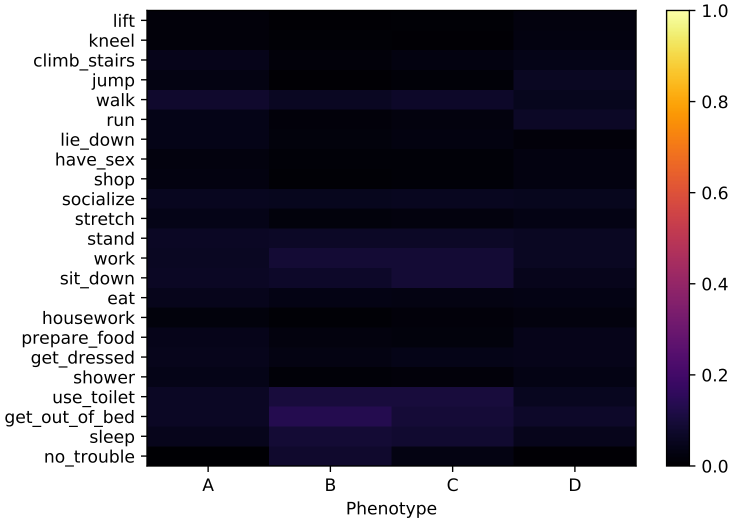

(k) Activities difficult to perform.

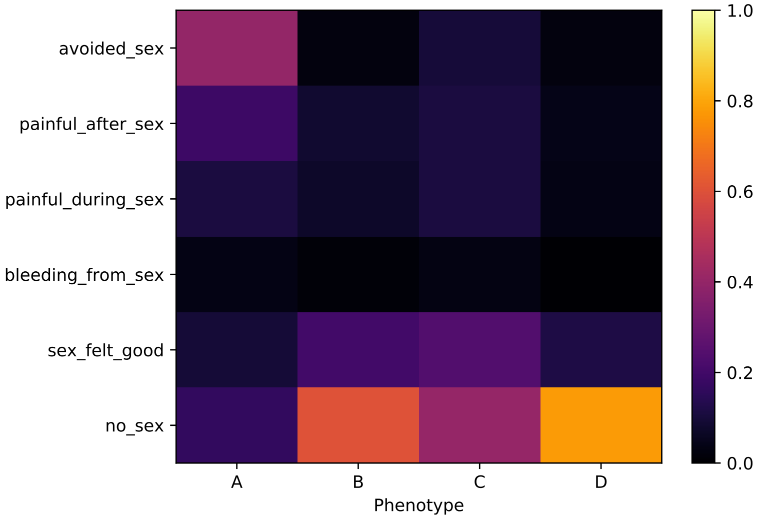

(l) Describe sex.

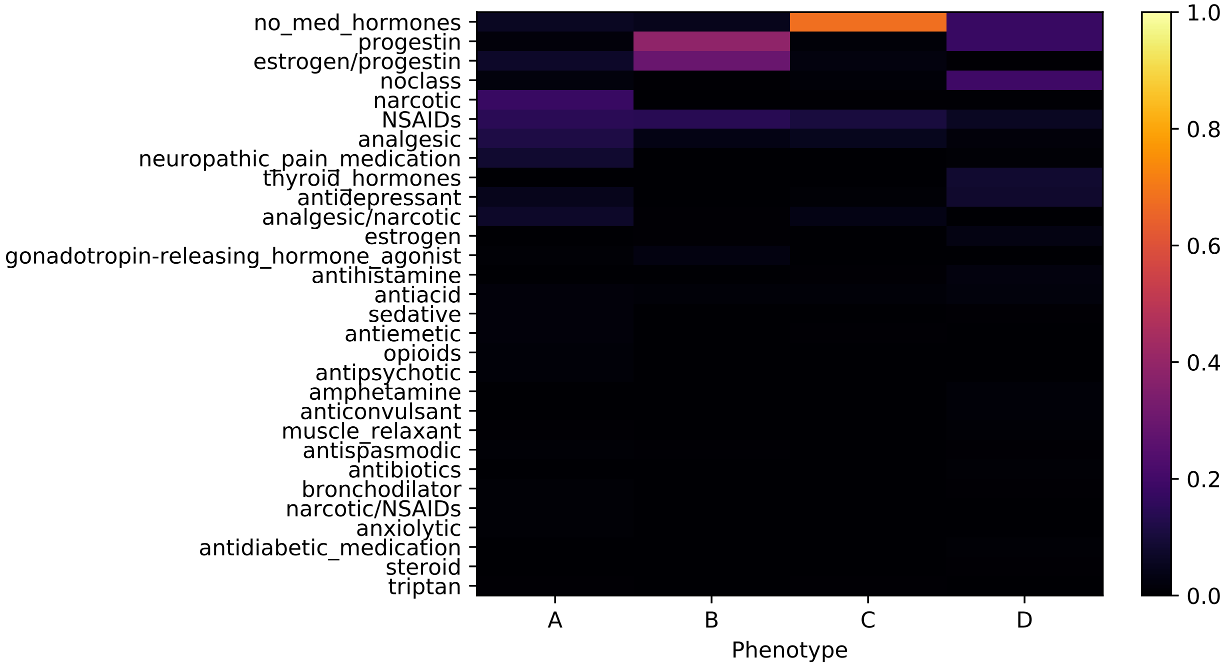

(m) Medications/hormones taken. For visual clarity, only the top 30 (most likely) vocabulary items of the posterior are displayed.

Supplementary Figure 1: Visualization of learned posteriors for endometriosis phenotypes. Each phenotype is defined as a set of per-question probability distributions across the answers to each of the thirteen questions. Each heatmap represents the likelihood of the answers within a question for a given phenotype. For instance, the "*no_ sex*" answer is highly likely to be tracked under phenotype D, and not likely to be tracked under phenotype A —yellow versus purple respectively, in heatmap (l).

1.3 Per-question answer-clouds

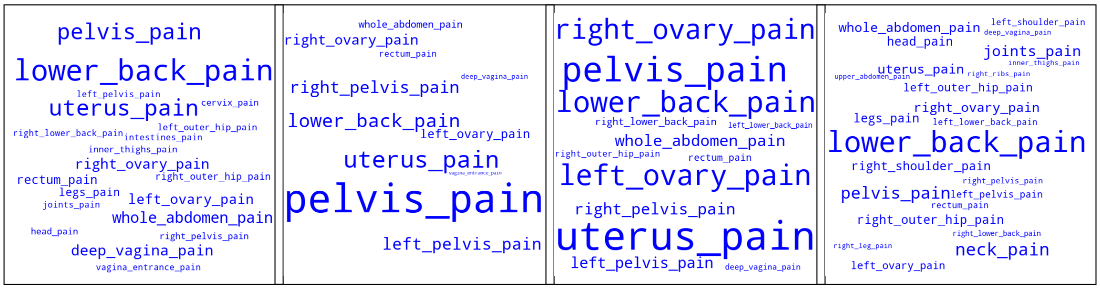

(a) Phenotype A (b) Phenotype B (c) Phenotype C (d) Phenotype D

Supplementary Figure 2: Where is the pain.

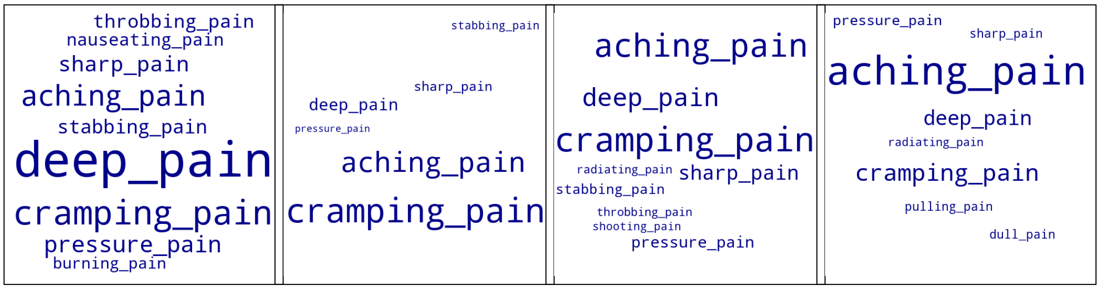

(a) Phenotype A (b) Phenotype B (c) Phenotype C (d) Phenotype D

Supplementary Figure 3: Describe the pain.

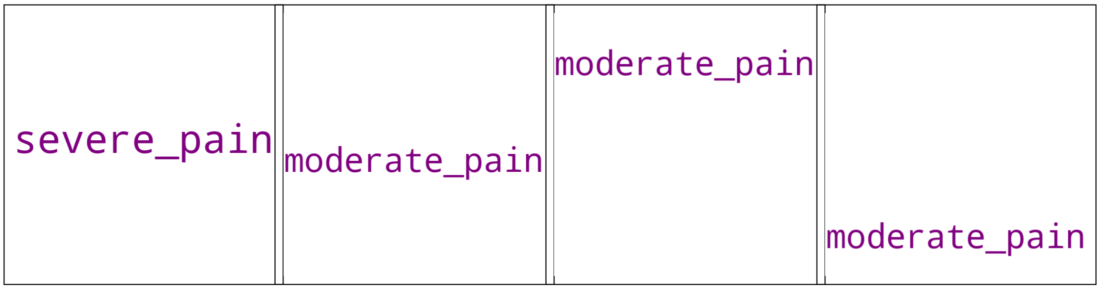

(a) Phenotype A (b) Phenotype B (c) Phenotype C (d) Phenotype D

Supplementary Figure 4: How severe is the pain.

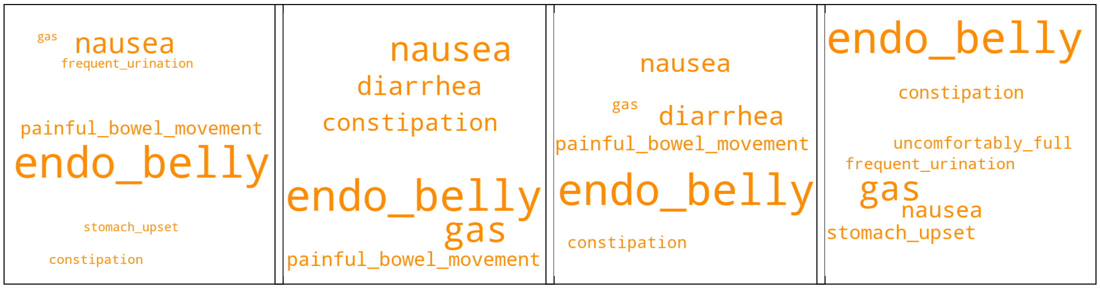

(a) Phenotype A (b) Phenotype B (c) Phenotype C (d) Phenotype D

Supplementary Figure 5: Describe GI/GU system.

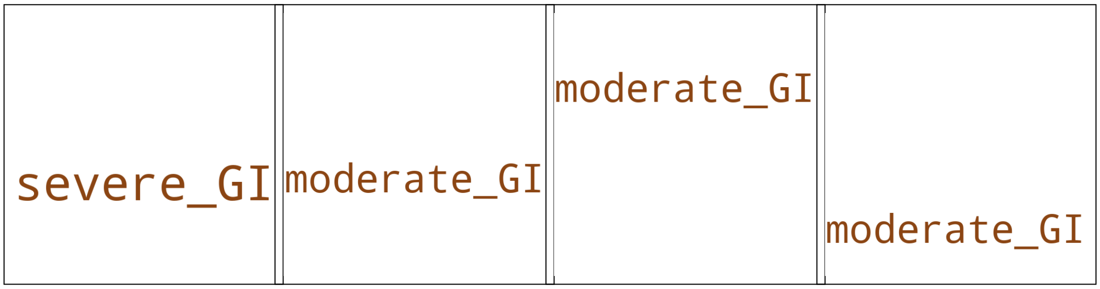

(a) Phenotype A (b) Phenotype B (c) Phenotype C (d) Phenotype D

Supplementary Figure 6: How severe is it.

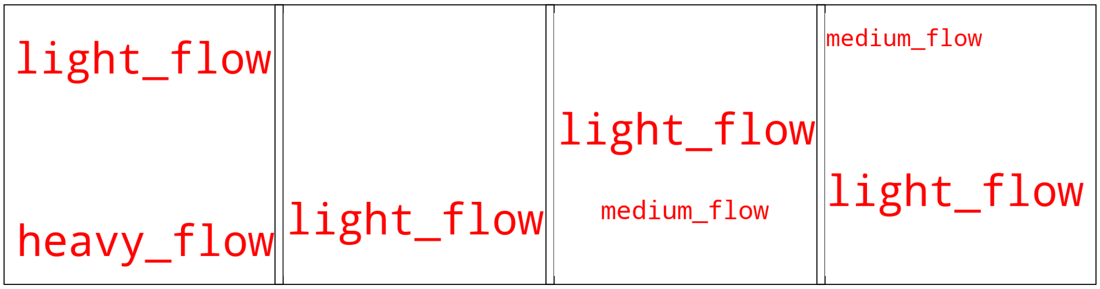

(a) Phenotype A (b) Phenotype B (c) Phenotype C (d) Phenotype D

Supplementary Figure 7: Describe your period flow.

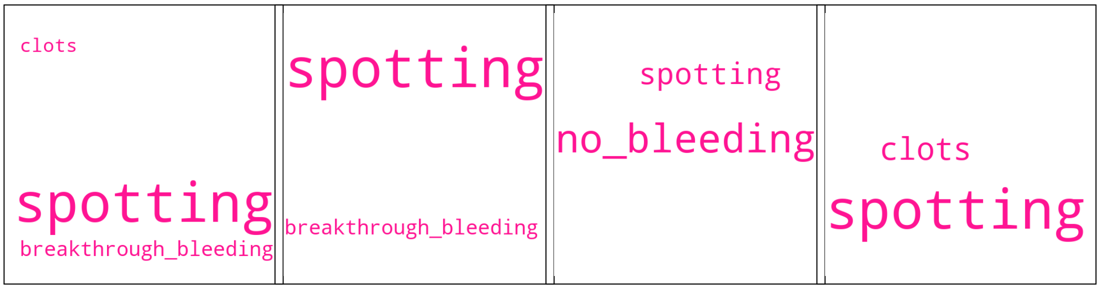

(a) Phenotype A (b) Phenotype B (c) Phenotype C (d) Phenotype D

Supplementary Figure 8: What kind of bleeding.

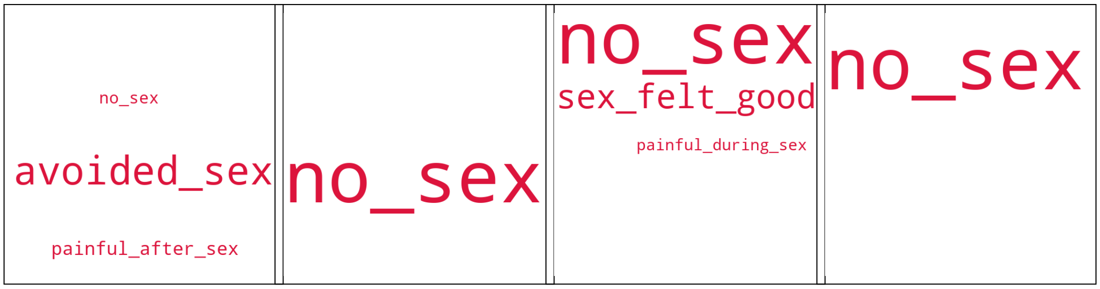

(a) Phenotype A (b) Phenotype B (c) Phenotype C (d) Phenotype D

Supplementary Figure 9: Describe sex.

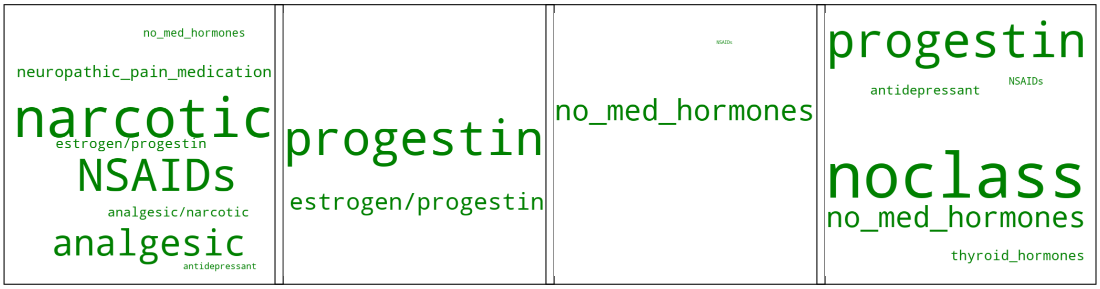

(a) Phenotype A (b) Phenotype B (c) Phenotype C (d) Phenotype D

Supplementary Figure 10: Medications/hormones taken.

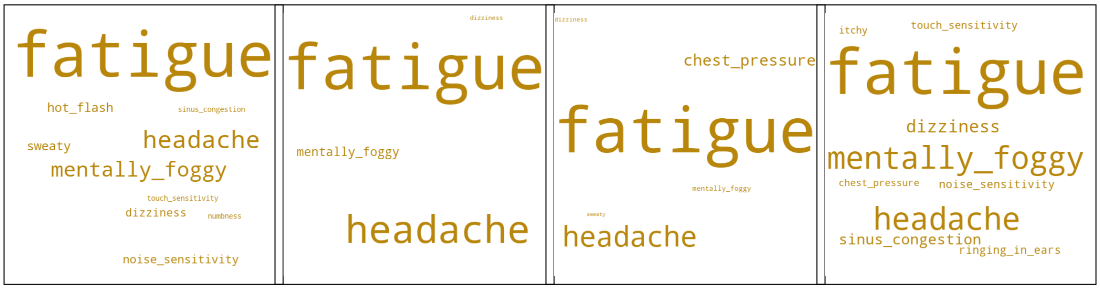

(a) Phenotype A (b) Phenotype B (c) Phenotype C (d) Phenotype D

Supplementary Figure 11: What are you experiencing.

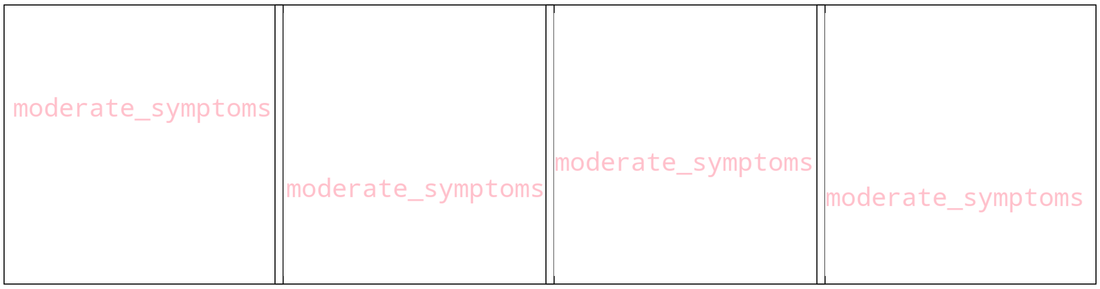

(a) Phenotype A (b) Phenotype B (c) Phenotype C (d) Phenotype D

Supplementary Figure 12: How severe is the symptom.

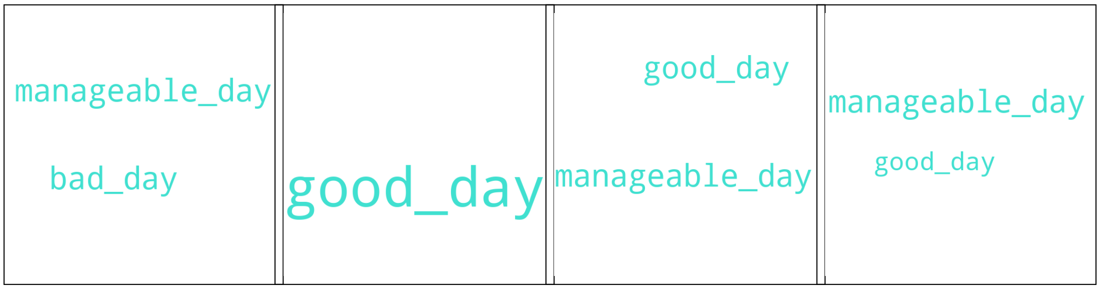

(a) Phenotype A (b) Phenotype B (c) Phenotype C (d) Phenotype D

Supplementary Figure 13: How was your day?

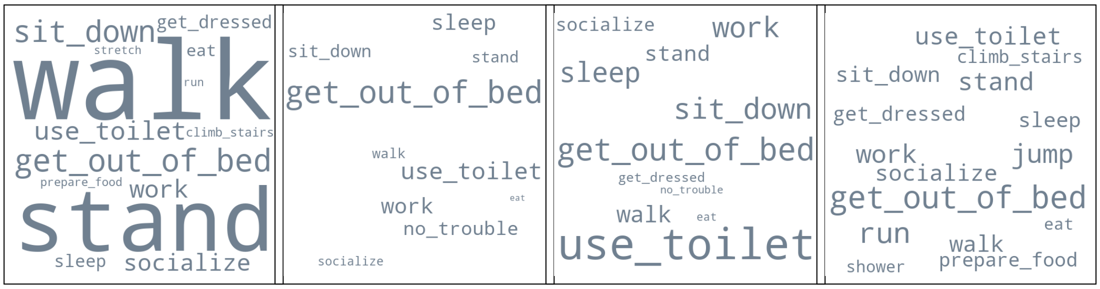

(a) Phenotype A (b) Phenotype B (c) Phenotype C (d) Phenotype D

Supplementary Figure 14: Activities difficult to perform

2 Supplementary Methods

2.1 K-means baseline clustering

A plausible baseline clustering approach is to group the available data in the Euclidean space formed by the concatenation of all the per-question response frequencies. That is, to count the number of times each user self-tracks a vocabulary item, and to form a count-based (or the corresponding normalized density) based feature vector. For this approach, the collection of responses to different questions $q = 1, \cdots, Q$ with their vocabulary size V_q is concatenated.

We find that a K-means based clustering in such $(V_1 + V_2 + \cdots + V_Q)$-dimensional Euclidean space does not attain any insightful endometriosis phenotypes: only two clusters dominate the groupings (see Supplementary Figure 15), and they provide no clinically meaningful associations with the WERF questionnaire responses. Besides, such an approach is hard to interpret clinically, as it is based on distances in a very high dimensional Euclidean space —the input is a $(V_1 + V_2 + \cdots + V_Q)$-dimensional vector.

An alternative baseline is to first reduce the input feature dimensionality —e.g., learn a map to a lower (2 or 3) dimensional space— and then cluster in this embedded space. We have successfully identified, via K-means, 4 separate cluster in such embedded space (shown in Supplementary Figure 15) both based on linear and non-linear dimensionality reduction techniques. However, none of the learned phenotypes are discriminative on clinically meaningful features. We find that many learned K-means clusters are associated with clinically meaningful outcomes (such as daily living, overall health or number of laparoscopies), and are therefore not discriminative on endometriosis relevant responses.

Besides, the interpretability of the learned phenotypes is hindered by the dimensionality reduction technique used. For example, if using the nonlinear t-Distributed Stochastic Neighbor Embedding (t-SNE) method that attains the most discriminative separation, one is faced with two challenges: (i) that as t-SNE optimizes a non-convex objective function via randomly initialized gradient descent algorithms, different runs result in different solutions; and (ii) that t-SNE does not retain distances from original to embedded spaces, resulting in uninterpretable original high-dimensional Euclidean distances.

On the contrary, we propose an extended mixed-membership model, which is a Bayesian generative model that can accommodate the inherent heterogeneity and uncertainty of the data, to capture the latent structure of collections of groups of self-tracked signs and symptoms.

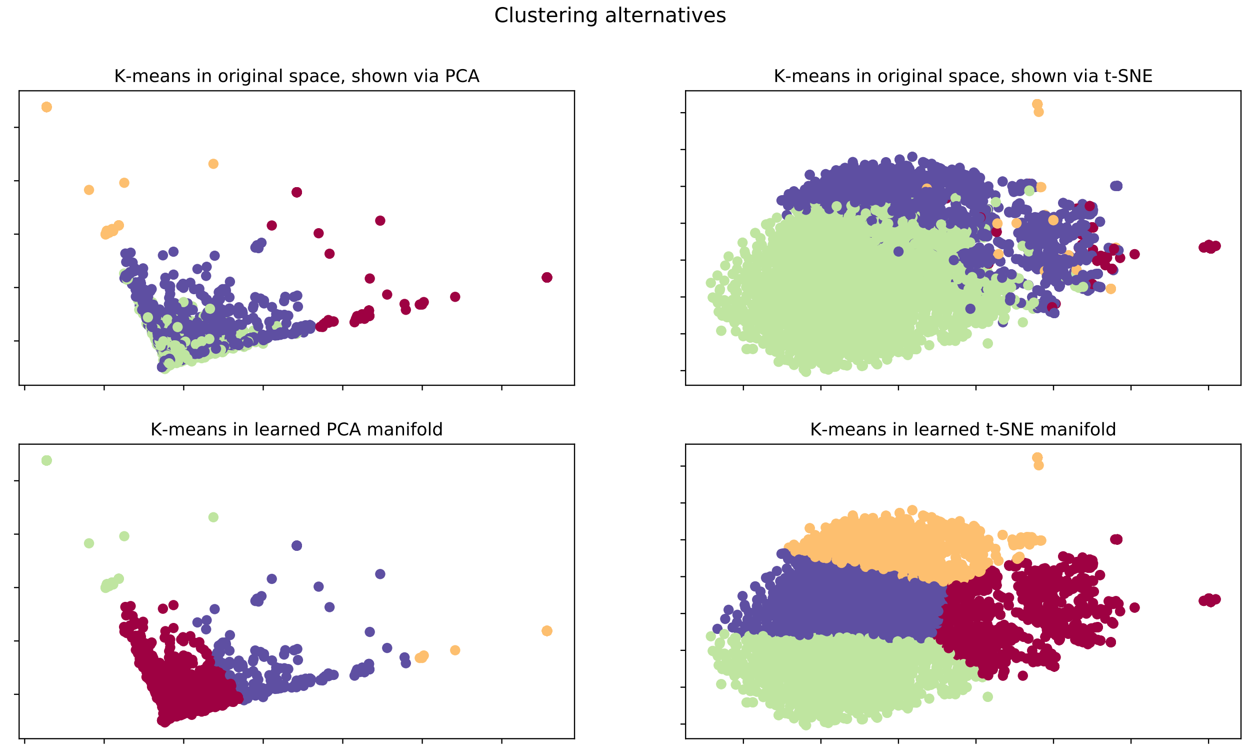

Supplementary Figure 15: K-means based clustering results: (top left) K-means in original space, shown in 2-d embedding learned by PCA; (top righ) K-means in original space, shown in 2-d embedding learned by t-SNE; (bottom left) K-means in embedded 2-dimensional space learned via PCA; (bottom right) K-means in embedded 2-dimensional space learned via t-SNE.

2.2 Extended Mixed membership model

The proposed extended mixed-membership model is illustrated in Supplementary Figure 16 and described below.

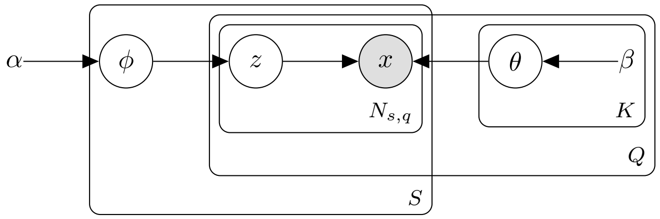

Supplementary Figure 16: Probabilistic graphical model of the proposed mixed-membership model.

The per-question mixed-membership generative process for subjects $s = 1, \cdots, S$ follows:

1. Draw per-subject phenotypic proportions $\phi_s \sim \text{Dirichlet}_K(\phi|\alpha)$ with hyperparameter α.
2. Draw per-phenotype and per-question response proportions $\theta_{k,q} \sim \text{Dirichlet}_{V_q}(\theta|\beta_{k,q})$, for all phenotypes $k = 1, \cdots, K$, and questions $q = 1, \cdots, Q$ with vocabulary size V_q, and hyperparameters $\beta_{k,q}$.
3. Draw per-subject observation phenotype assignments $z_{s,n} \sim \text{Categorical}_K(z|\phi_s)$, for $n = 1, \cdots, N_s$.
4. Draw per-subject questions responses $x_{s,n}|z_{s,n}, q_{s,n} \sim \text{Categorical}_{V_{q_{s,n}}}(x|\theta_{z_{s,n},q_{s,n}})$, for $n = 1, \cdots, N_s$, where $q_{s,n}$ indicates the response n to question q by subject s.

After observing a data-set with N_{sq} responses per-subject and question, the goal is to infer the phenotypic proportions ϕ_s and the set of K phenotypes parameterized by $\theta_{k,q}$ per-question. To that end, and due to the conjugacy assumptions in the generative process, we resort to a collapsed Gibbs sampler that utilizes the following distribution

$$p(z_{s,n^*} = k|x_{s,n*}, q_{s,n*}, \alpha_N, \beta_N) \propto p(z_{s,n^*} = k|\alpha_{s,N})p(x_{s,n^*}|q_{s,n^*}, z_{s,n^*}, \beta_{k,q,N}) \; , \\ \text{with} \begin{cases} p(z_{s,n^*} = k|\alpha_{s,N}) = \frac{\alpha_{k,s,N}}{\sum_{k=1}^{K} \alpha_{k,s,N}} \; , \\ p(x_{s,n^*}|q_{s,n^*}, z_{s,n^*}, \beta_{k,q,N}) = \frac{\beta_{k,q,v_q,N}}{\sum_{v_q=1}^{V_q} \beta_{k,q,v_q,N}} \; , \end{cases} \tag{1}$$

where the parameters for the updated posteriors are

$$\begin{aligned} p(\phi_s|Z_N, \alpha_0) &= \text{Dirichlet}_K(\phi_s|\alpha_{s,N}) \; , \qquad \text{with } \alpha_{k,s,N} = \alpha_{k,0} + N_{s,k} \; ; \\ p(\theta_{k,q}|X_N, Q_N, Z_N, \beta_{k,q,0}) &= \text{Dirichlet}_{V_q}(\theta_{k,q}|\beta_{k,q,N}) \; , \qquad \text{with } \beta_{k,q,v_q,N} = \beta_{k,q,v_q,0} + N_{k,q,v_q} \; . \end{aligned} \tag{2}$$
